# A m^6^A regulators-related classifier for prognosis and tumor microenvironment characterization in hepatocellular carcinoma

**DOI:** 10.3389/fimmu.2024.1374465

**Published:** 2024-07-25

**Authors:** Shaohua Xu, Yi Zhang, Ying Yang, Kexin Dong, Hanfei Zhang, Chunhua Luo, Song-Mei Liu

**Affiliations:** ^1^ Department of Clinical Laboratory, Center for Gene Diagnosis & Program of Clinical Laboratory, Zhongnan Hospital of Wuhan University, Wuhan, China; ^2^ The First College of Clinical Medical Science, China Three Gorges University, Yichang, China

**Keywords:** N^6^-methyladenosine, WGCNA, SVM-RFE, LASSO, consensus clustering algorithm, TIICs, DCA

## Abstract

**Background:**

Increasing evidence have highlighted the biological significance of mRNA N^6^-methyladenosine (m^6^A) modification in regulating tumorigenicity and progression. However, the potential roles of m^6^A regulators in tumor microenvironment (TME) formation and immune cell infiltration in liver hepatocellular carcinoma (LIHC or HCC) requires further clarification.

**Method:**

RNA sequencing data were obtained from TCGA-LIHC databases and ICGC-LIRI-JP databases. Consensus clustering algorithm was used to identify m^6^A regulators cluster subtypes. Weighted gene co-expression network analysis (WGCNA), LASSO regression, Random Forest (RF), and Support Vector Machine-Recursive Feature Elimination (SVM-RFE) were applied to identify candidate biomarkers, and then a m^6^Arisk score model was constructed. The correlations of m^6^Arisk score with immunological characteristics (immunomodulators, cancer immunity cycles, tumor-infiltrating immune cells (TIICs), and immune checkpoints) were systematically evaluated. The effective performance of nomogram was evaluated using concordance index (C‐index), calibration plots, decision curve analysis (DCA), and receiver operating characteristic curve (ROC).

**Results:**

Two distinct m^6^A modification patterns were identified based on 23 m^6^A regulators, which were correlated with different clinical outcomes and biological functions. Based on the constructed m^6^Arisk score model, HCC patients can be divided into two distinct risk score subgroups. Further analysis indicated that the m^6^Arisk score showed excellent prognostic performance. Patients with a high m^6^Arisk score was significantly associated with poorer clinical outcome, lower drug sensitivity, and higher immune infiltration. Moreover, we developed a nomogram model by incorporating the m^6^Arisk score and clinicopathological features. The application of the m^6^Arisk score for the prognostic stratification of HCC has good clinical applicability and clinical net benefit.

**Conclusion:**

Our findings reveal the crucial role of m^6^A modification patterns for predicting HCC TME status and prognosis, and highlight the good clinical applicability and net benefit of m^6^Arisk score in terms of prognosis, immunophenotype, and drug therapy in HCC patients.

## 1 Introduction

Hepatocellular carcinomas (HCC, accounting for 90% of liver cancer) is one of the most frequent fatal malignancies and ranks fourth among cancer-related mortality worldwide (1). Despite recent great advances in treatment interventions, 5-year overall survival (OS) for HCC patients remains poor and unsatisfactory, with only 5% to 15% of early-stage patients qualifying for surgical excision (2). HCC is insidious and develops rapidly, and patients are usually diagnosed at an advanced stage. The treatment strategies that are currently available for more than 90% of liver cancer patients mainly include chemotherapy, immunotherapy, natural compounds, and nanotechnology (2). However, the clinical benefit of these therapies remains unsatisfactory, mainly due to the lack of effective pre-treatment predictive biomarkers. Besides, treatment of regional resection and liver transplantation is still limited, and the recurrence rate after regional resection is high. Therefore, it is imperative to identify novel reliable biomarkers and therapeutic targets that enable early diagnosis and treatment response prediction for HCC patients.

Although the risk factors for liver carcinogenesis are well defined (including hepatitis B and C viruses, fatty liver, alcoholic cirrhosis, diabetes, obesity, etc), the underlying molecular mechanisms remain ambiguous. Extensive evidence shows that epigenetic mechanisms is implicated in multiple aspects of cancer biology, from driving primary tumor growth and invasion to modulating the immune response within the tumor microenvironment (TME). The complex bidirectional dynamic cross-talk between cancer cells and their microenvironment has been identified as a key factor that drives tumor initiation, growth, progression, malignant conversion, invasion, metastasis, drug resistance and patient prognosis (3–5). TME is a complex and evolving multi-layered cellular environment composed of stroma, vascular, and innate/adaptive immune cells, as well as a community of malignant clones (6). N^6^-methyladenosine (m^6^A) methylation is one of the most common types of modifications in eukaryotic messenger RNA (mRNA). Similar to modifications in DNA or proteins, it is regulated by various types of regulators, including methyltransferases (“ writers “), RNA-binding proteins (“ readers “), and demethylases (“ erasers “). Dysregulation of m^6^A regulatory factors is associated with malignant tumor progression and TME-specific immunomodulation abnormalities (7, 8). Nonetheless, the role of m6A regulators in TME heterogeneity and immune cell infiltration in HCC remains to be further investigated. Therefore, it is crucial to comprehensively understand the relationship between RNA methylation modification patterns and genetic alterations underlying cancer cell heterogeneity.

Cancer is both a genetic and epigenetic disease. Gene mutations and epigenetic alterations have been identified as significant contributors to human carcinogenesis. Unlike genetic mutations, epigenetic modifications refer to heritable changes that mediate gene expression without altering the genetic DNA sequence (9). Extensive evidence shows that epigenetic mechanisms is implicated in multiple aspects of cancer biology, from driving primary tumor growth and invasion to modulating the immune response within the TME. Epigenetics-based diagnostic and prognostic tools also greatly contribute to the development of precision oncology. Recent studies have reported that abnormal decreases or increases in the overall abundance of m^6^A in some types of cancer may be associated with cancer progression and clinical outcomes. It has been reported that the overall abundance and expression level of m^6^A in mRNA or total RNA in human gastric cancer and liver cancer tissues are significantly increased, and are closely related to the expression level of m^6^A methylation regulatory enzymes (10, 11). It has also been reported that the overall abundance of m^6^A is significantly reduced in more advanced human bladder cancer tissues and is associated with poor prognosis in bladder cancer patients (12). Another study showed that m^6^A abundance is associated with therapeutic drug response and may be an epigenetic driver of chemotherapy resistance (13). Together, these results suggest that m^6^A modification regulators have different potential in prognosis stratification and the development of new therapeutic strategies across various cancers. Due to immune evasion and heterogeneity in the TME, only a minority of patients respond favorably to immunotherapy. At this point, better stratification is urgently needed for HCC patients to enhance treatment efficacy. Therefore, comprehensive investigation of m^6^A modification and its biological roles in HCC may contribute to improving prognosis prediction and personalized precision treatment approaches for HCC.

In this study, we first profiled the expression of 23 m^6^A regulators and identified two distinct m^6^A regulator-mediated modification patterns based on TCGA-LIHC cohort. We then constructed a novel m^6^A-risk scoring system to quantify the m^6^A modification patterns in individual tumors and to predict the clinical response of HCC patients to common chemotherapy or targeted drugs. Additionally, we comprehensively evaluated the association between m^6^A modification patterns and TME cell-infiltrating characteristics.

## 2 Materials and methods

### 2.1 Data source and preprocessing

RNA-sequencing data (counts value) with corresponding complete clinical information of HCC were obtained from TCGA-LIHC program (https://portal.gdc.cancer.gov/repository) and ICGC-LIRI-JP database (https://dcc.icgc.org). The annotation file of GRCh38 (version 36) was downloaded from GENCODE to identify the length of each mRNA. Subsequently, RNA-sequencing data in counts format was transformed into transcripts per kilobase million (TPM) format and further subjected to log2 transformation for normalization. In addition, somatic mutation data and CNV files were retrieved from the TCGA-LIHC program. Samples lacking clinicopathological information or survival outcomes were excluded from further analysis. Ultimately, 23 acknowledged m^6^A regulator genes, including 8 writers, 13 readers, and 2 erasers, were identified from previous studies (14–16).

### 2.2 Unsupervised clustering of m^6^A regulator genes

Consensus unsupervised clustering analysis was employed for identifying distinct m^6^A regulator modification patterns in the TCGA-LIHC cohort by the k-means algorithms, which is available in the “ConsensusClusterPlus” R package (17, 18). The “ConsensusClusterPlus” package provides quantitative stability evidence to determine a cluster count and cluster membership in an unsupervised analysis. The quantity and stability of clusters were determined by consensus clustering algorithm, and conducted for 1,000 iterations (18). The cumulative distribution function (CDF) curves were used to determine the optimal number of clusters, indexed by k-means algorithms value from 2 to 9. Ultimately, based on the clustering effect, the clustering stability was higher when k = 2.

### 2.3 Differentially expressed genes analysis

The expression profile data from TCGA-LIHC cohorts were preprocessed by R software (V.4.0.5). The differential expression analysis between two distinct m^6^A cluster subtypes were performed using the “DESeq2” R package (19) (V.1.38.3). Genes with |log2FoldChange| > 1 and *P* adj < 0.001 were regarded as statistically significant. Furthermore, Gene ontology (GO) and Kyoto Encyclopedia of Genes and Genomes (KEGG) pathway enrichment analyses were performed for DEGs using the “clusterProfiler” R package. GO categories comprised biological processes (BP), molecular functions (MF), and cellular components (CC). The *p*-value was adjusted using the Benjamini–Hochberg (BH) approach or False Discovery Rate (FDR) for multiple testing corrections. The results satisfied FDR < 0.05 were regarded as statistically significant.

### 2.4 Gene set enrichment analysis

This analysis aimed to discern potentially relevant gene expression signatures between distinct m^6^A cluster subtypes utilizing the ‘clusterProfiler’ package (V.4.6.0). The reference gene set for GSEA analysis, ‘c2.cp.kegg.v7.4.symbols.gmt,’ was obtained from MSigDB database (http://software.broadinstitute.org/gsea/msigdb/index.jsp). Differential expression analysis between the two cluster subtypes was conducted using “DEseq2” package (V.1.38.3). Subsequently, all genes were ranked from high to bottom according to log2-fold change, and this sorted gene set was used for GSEA analysis. For achieving a normalized enrichment score (NES) for each analysis, a permutation test with 1,000 iterations were performed. The pathways meeting the criteria of |NES| > 1, *p*-value < 0.05, and *q*-value < 0.05 were regarded as significant enrichment.

### 2.5 Gene set variation analysis

This analysis was performed to assess the variation of hallmark pathway activity in distinct m^6^A cluster subtypes via ‘GSVA’ package (V.1.38.0) in an unsupervised manner (20). In this study, the gene set ‘h.all.v7.4.symbols.gmt’ was selected as the background gene set for GSVA analysis, which was downloaded from MSigDB database (21). The ‘limma’ R package was utilized to analyze the differences in hallmark pathways between two m^6^A cluster subtypes. The criteria for screening significant difference were as follows: |t-value| >2 and p-values < 0.05. The pathway with a t-value > 0 was thought to be activated in the m^6^A cluster B, and conversely, the pathway with a t-value < 0 was considered to be activated in the m^6^A cluster A.

### 2.6 Weighted gene co-expression network analysis

WGCNA R package was utilized to construct an unsigned weighted co-expression network to identify m^6^A cluster-related gene modules. First of all, TCGA-LIHC expression data in TPM format were evaluated for availability and genes were screened using the lowest median absolute deviation (MAD) for further analysis. The Pearson’s correlation matrices between all included genes were calculated, and then transformed into an unsigned weighted adjacency matrix using a power function. The power β was estimated by soft-threshold of 0.85 to obtain a network with scale-free topology. Furthermore, a topological overlap measure (TOM) matrix was generated to estimate the connectivity property of nodes in the network. The node in the networks represented a coding gene in the modules and an edge connecting two genes indicated a strong correlation. Average linkage hierarchical clustering was used to construct a clustering dendrogram of the TOM matrix. Dynamic tree-cutting algorithm was used to obtain appropriate modules of co-expressed genes with deep split = 2 and the minimum gene module size of 40, and the height cutting threshold of merging similar modules was set to 0.3. Genes outside of each module were denoted with color “grey”. The association between module Eigengenes (ME) values with clinicopathological characteristics was assessed by Pearson’s correlation, and the modules with the strongest association with m^6^A cluster were selected for further analysis.

### 2.7 Identification of optimal feature gene biomarkers

To identify the optimal feature gene variables with the superior discriminative power, three machine-learning algorithms were implemented to predict disease status, including LASSO (least absolute shrinkage and selection operator) regression, SVM-RFE (support vector machine-recursive feature elimination), and RF (random forest classifier). LASSO regression analysis was performed using the ‘glmnet’ R package (22), and SVM-RFE using the ‘e1071’ R package (23). In the LASSO regression analysis, the response type was configured as binomial, and the alpha parameter was set to 1. Meanwhile, SVM-RFE model was compared by the average mis-judgement rates of their 10-fold cross-validations (24). The final importance of features was based on the average importance of each feature variable in each iteration. In the RF algorithm, the importance ranking of each gene, and the error rate and accuracy rate of the combination in each iteration were obtained using the RFE method. The feature genes were the corresponding genes in the optimal combination with the lowest error rate. The overlapping genes between the three machine-learning algorithms were regarded as optimal diagnostic biomarkers. The accuracy of the overlapping genes for diagnosis was evaluated using the receiver operating characteristic curve (ROC) in TCGA-LIHC dataset, and the expression levels of candidate genes were further validated in the ICGC-LIRI-JP dataset.

### 2.8 Construction of m^6^Arisk score model for HCC prognosis

The overlapping feature genes obtained above were first subjected to univariate Cox regression to obtain the OS related DEGs. Followed by least absolute shrinkage and selection operator (LASSO) penalties regression, we identified the most powerful prognostic DEGs and their correlative coefficients using “glmnet” R package. Meanwhile, the “caret” R package was utilized to randomly divide the TCGA-LIHC cohort (n = 371) with a ratio of 1:1, with 50% of the data used for training and 50% for validation. Next, the independent prognostic feature genes were identified using multivariate Cox regression analysis to construct a m^6^A related prognostic risk score model in the training set. Then, the m^6^Arisk scores were calculated using the formula: m^6^Arisk-score = Σ (gene expression * risk coefficient). Based on the median of risk score, the training set and testing set were stratified into low- and high-risk groups, respectively. Finally, survival analysis and receiver operating characteristic (ROC) curve analysis were carried out for the two risk groups using the “survminer” and “survivalROC” R packages, respectively.

### 2.9 The immunological characteristics of the tumor microenvironment

To confirm the role of m^6^Arisk score in modulating cancer immunity in HCC, we analyzed the correlation between m^6^Arisk and the immunological characteristics of TME. The immunological characteristics included the activity of the cancer immunity cycle, infiltration level of tumor‐infiltrating immune cells (TIICs), and the expression of immunomodulators and inhibitory immune checkpoints. The cancer immunity cycle consists of seven steps that reflect the anticancer immune response and determine the fate of the tumor cells (25) (**Supplementary Table S12**). The immunomodulators comprise major histocompatibility complex (MHC), receptors, chemokines, and immune stimulators (26) (**Supplementary Table S17**). In this study, the activities of the cancer immunity cycle were also quantified using a single sample gene set enrichment analysis (ssGSEA) as previously reported (27). Moreover, to avoid the calculation error of different algorithms and marker gene sets, six independent algorithms [including Cibersort (28), MCP-counter (29), quanTIseq (30), TIMER (31), xCell (32), and TISIDB (33)] were used to comprehensively calculate TIICs infiltration level in TME (**Supplementary Table S7**). Thereafter, the effector genes of TIICs and inhibitory immune checkpoints were also identified and collected from previous studies (34) (**Supplementary Tables S18**, **S19**).

### 2.10 Somatic mutation analysis

For genomic layer analysis, the mutation annotation format (MAF) data of HCC patients was derived from the TCGA-LIHC database (http://tcga-data.nci.nih.gov/tcga/) and analyzed using the “maftools” R package (35). The mutation profile was visualized using a waterfall plot, which displays the mutation types and frequencies of the top driver genes. Fisher’s exact test was conducted to compare the differential mutation patterns between the two distinct m^6^Arisk score groups. Genes with a *p*-value less than 0.05 were considered statistically significant and were visualized in a forest plot. In addition, a lollipop diagram was drawn to indicate the mutation types of the most frequently mutated gene in order to provide insight into the molecular alterations associated with hepatocellular carcinoma (HCC) development. Furthermore, the exclusivity and co-occurrence of mutations for the top 20 mutated genes were analyzed. The prognostic value of TMB and the combination of TMB and m^6^Arisk scores were comprehensively evaluated. Additionally, the relationship between the m^6^Arisk scores and the cancer stem cell (CSC) index was evaluated to investigate their potential association in tumor progression and treatment resistance.

### 2.11 Prediction of therapeutic response by m^6^Arisk score

The T cell receptor (TCR) repertoire is a well-characterized immune trait that plays a key role in the selective activation of the adaptive immune system (36, 37), tightly linked to the immune status and anti-tumor immune response. In this study, we obtained the TCR Shannon diversity index and richness of the TCGA-LIHC cohort from previous literature (36) and investigated their differences between the two distinct m^6^Arisk scores groups. The Tumor Inflammation Signature (TIS) is a transcriptome-based algorithm consisting of 18 genes that measures a pre-existing but suppressed adaptive immune response within the tumor (38). We computed the TIS score of each patient as previously reported (39) in TCGA-LIHC dataset to speculate on the association between m^6^Arisk scores and the adaptive immune response. Imunophenoscore (IPS), a machine learning-based scoring scheme that represents the determinants of immunogenicity, has been proven to be tightly linked to the survival of multiple cancer and is a promising predictor of response to immunotherapy (26). We obtained the IPS of HCC from the Cancer Immunome Atlas (TCIA) (https://tcia.at/home) and compared them between the two m^6^Arisk-score groups to predict the immunotherapeutic sensitivities.

Moreover, to explore the potential clinical applications of the m^6^Arisk score in treatment decisions, we utilized the “oncoPredict” R package (40) to infer the semi-inhibitory concentration (IC50) values of commonly used targeted/chemotherapy drugs. We then performed a correlation analysis between the IC50 values and the m^6^Arisk-score groups using the Wilcoxon test. The drugs and their target information were derived from DrugBank (https://go.Drugbank.com/). This analysis aimed to investigate the relationship between m^6^Arisk score and the response to specific drugs, providing insights into personalized treatment strategies.

### 2.12 Establishment and validation of a nomogram scoring system

The m^6^Arisk scores and common clinical variables (including age, gender, and TNM stages) were incorporated to establish a nomogram scoring system using the “rms” R package (41). In this study, the time-dependent ROC curves of nomogram and clinical prognostic variables at 1-, 3-, and 5-year were generated, and the corresponding time-dependent area under the curves (AUCs) was calculated to evaluate the discrimination of nomogram. The calibration curves and the decision curve analysis (DCA) of 1-, 3-, and 5-year were plotted to assess the prediction accuracy and clinical net benefit of nomogram, respectively (42, 43). In addition, concordance index (C-index) was also performed to assess the prediction efficiency and accuracy of nomogram. A C-index score around 0.70 indicates a good model, whereas a score around 0.50 suggests random background.

### 2.13 Clinical sample collection, RNA isolation, and qPCR

Twenty-eight pairs of fresh-frozen tissues (HCC tissues and adjacent tissues) were collected from the Zhongnan Hospital of Wuhan University and approved by the ethics committee (Approval Number 2017058). Written informed consent was obtained from all the participants. Complementary DNA (cDNA) was synthesized from total RNA using the Prime Script RT Reagent Kit (Vazyme, R333-01, China). The SYBR Prime Script RT-PCR kit (Vazyme, Q712-02, China) was used for qPCR on a CFX96 instrument (Bio-Rad, America). Gene expression levels were calculated with the 2^-ΔΔct^ strategy and normalized to the “housekeeping” gene β-actin. The primer sequences were integrated into **Supplementary Table S20**.

### 2.14 Statistical analysis

All statistical analyses and graphical plotting were performed using R software (version 4.0.5.) Unless stated otherwise, *P <*0.05 (two-sided) was considered statistically significant.

## 3 Results

### 3.1 Landscape of genetic variation of 23 m^6^A regulators in LIHC

**Figure 1:**
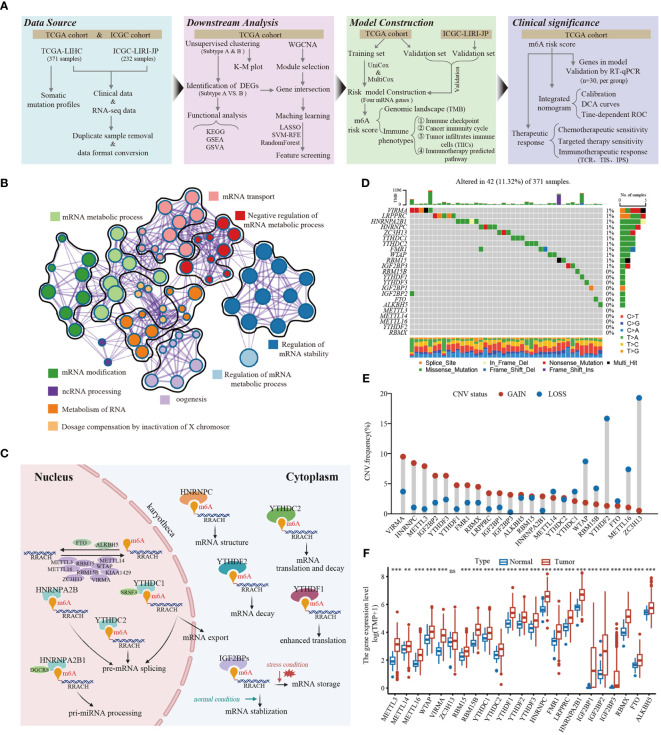
The landscape of genetic and transcriptional alterations of m6A regulators in HCC. **(A)** The schematic workflow of this study. K-M plot, Kaplan-Meier plot; GSEA, gene set enrichment analysis; GSVA, gene set variation analysis; WGCNA, weighted gene co-expression network analysis; ROC, receiver operating characteristic; LASSO, least absolute shrinkage and selection operator; SVM-RFE, support vector machine recursive feature elimination; UniCox, univariate Cox; MultiCox, multivariate Cox; DCA, decision curve analysis, TCR, T cell receptor; TIS, Tumor Inflammation Signature; IPS, Imunophenoscore. **(B)** The enrichment network of 23 m^6^A regulators visualized by Metascape (https://metascape.org/), showed the similarity of enrichment terms within and between clusters. **(C)** The regulation mechanism of m^6^A “writer,” “eraser,” and “reader” proteins on RNA metabolism. **(D)** Mutation frequencies of 23 m^6^A regulators in 371 HCC patients from TCGA-LIHC cohort. **(E)** Frequencies of copy number variant (CNV) of the 23 m^6^A regulators. **(F)** The differential expression levels of 23 m^6^A regulators between tumor and normal tissues. ** *P* < 0.01; *** *P* < 0.001; ns, No significance.

In this study, we identified 23 m^6^A RNA methylation regulatory genes (including eight “writers,” thirteen “readers,” and two “erasers”) from the published literature, and systematically investigated the roles of them in LIHC. The workflow for this study is shown in **Figure 1A**. Additionally, the significantly enriched biological processes of the 23 m^6^A regulators were summarized using the Metascape database, as depicted in **Figure 1B**. These processes primarily revolve around mRNA stability, mRNA transport, mRNA metabolic processes, mRNA modification, and ncRNA processing. **Figure 1C** illustrates the dynamic reversible process of the m^6^A regulators, showcasing their ability to recognize, remove, and add m^6^A-modified sites. These analyses provided insights into the regulatory complexity and functional implications of m^6^A RNA methylation in gene expression and RNA metabolism. The somatic mutations analysis of 23 m^6^A regulators demonstrated that a total of 42 of the 371 (11.3%) TCGA-LIHC samples experienced genetic alterations of m^6^A regulators, primarily including missense mutations and splice site (**Figure 1D**). Moreover, the CNA analysis revealed CNV alterations were prevalent in the 23 m^6^A regulators, with most of the alterations being focused on gene amplification (such as *VIRMA*, *METTL3*, *HNRNPC*, *IGF2BP2*, and *YTHDF3*), whereas *WTAP*, *YTHDF2*, and *ZC3H13* showed the highest deletion frequency (**Figure 1E**). Further investigation of the expression profiles of the 23 m^6^A regulators indicated that most of the m^6^A writers (*METTL3/14/16*, *WTAP*, *VIRMA*, and *RBM15/15B*), readers (*YTHDC1/2*, *YTHDF1/2/3*, *HNRNPC*, *FMR1*, *LRPPRC*, *HNRNPA2B1*, *IGF2BP1/2/3*, and *RBMX*), and erasers (*FTO* and *ALKBH5*) were markedly upregulated in the tumor tissues (**Figure 1F**). The survival analysis revealed that most of the m^6^A regulators were significantly correlated with LIHC prognoses (**Supplementary Figure S1**). Taken together, these results demonstrate that m^6^A regulators may act as diagnostic biomarkers and prognostic predictors for LIHC.

### 3.2 Identification of m^6^A modification subtypes and function enrichment analysis

**Figure 2:**
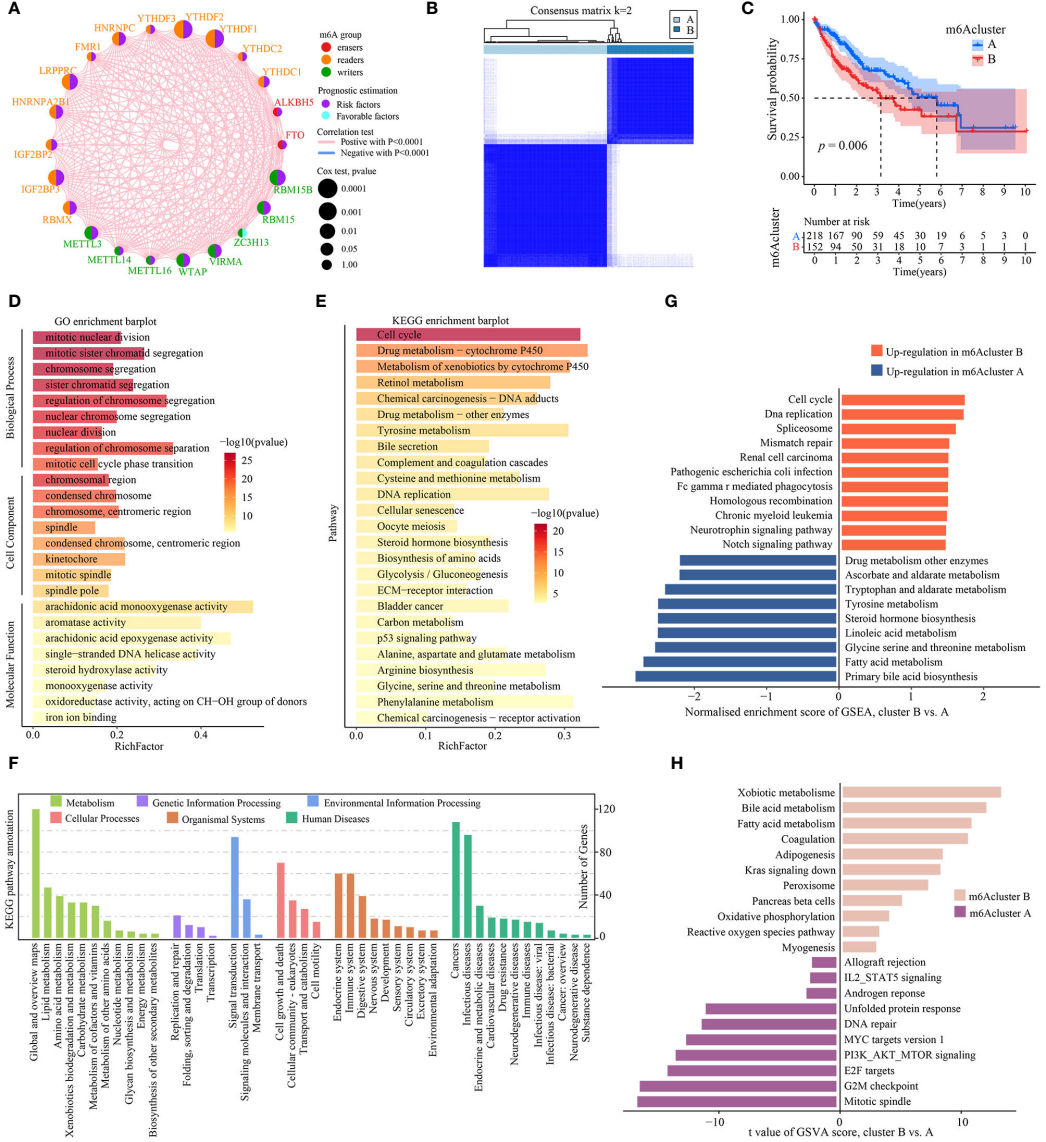
Identification and functional enrichment analysis of m^6^A cluster subtypes. **(A)** The interaction analysis of expression on 23 m^6^A regulators in TCGA-LIHC. Different colored circles represent different modification types of m6A regulators. The size of the circle represents the prognostic effect of each m^6^A regulator and scaled by *p* value. Connecting lines represent interactions between each other. **(B)** The consensus score matrix of 371 samples (k = 2). **(C)** Kaplan‐Meier curves for estimating the overall survival between subtypes of m^6^A cluster. **(D)** GO enrichment and **(E)** KEGG enrichment analyses of the DEGs (|log2FoldChange| > 1, *P*-adj < 0.001) between m6A cluster B and A. The top 25 enriched terms are shown. The color of the bars denotes the negative logarithm of the p-value of the hypergeometric test. **(F)** The bar charts showing KEGG pathway annotation. The color indicates the category A of annotation terms. The horizontal coordinate presents the category B of annotation terms, and the ordinate denotes the number of genes (hits) of category B. **(G)** Bar charts showing the top 10 KEGG pathway terms enriched by GSEA. Red and blue represent the upregulated pathway terms in m^6^A cluster B and A, respectively. **(H)** The GSVA score of hallmark pathway activities curated from MSigDB in distinct m^6^A modification patterns. T values are from two-sided unpaired limma-moderated t test (linear models), corrected for effects from the patient of origin.

**Figure 2A** presented the interactions and interconnections among the 23 m^6^A regulators and their prognostic value in TCGA-LIHC patients. Most of these genes were risk factors and were significantly positively correlated with each other (*p*<0.001). The results suggested that the cross-talk between these m^6^A regulators probably play important roles in the formation of different modification patterns and was implicated in the pathogenesis and progression of tumor. To further explore the modification patterns of m^6^A regulators, unsupervised clustering algorithms based on the expression profiles of 23 m^6^A regulators were applied to construct m^6^A subtypes. As shown in **Figure 2B** and **Supplementary Figure S2**, the consensus score matrix revealed that k = 2 appeared to be an optimal choice for ensuring the least crossover between TCGA-LIHC samples. Next, Kaplan-Meier survival curves showed that m^6^A cluster A presented significantly better prognoses than cluster B (*P* = 0.006; **Figure 2C**).

Next, the representative DEGs (|log2FoldChange| > 1, *P*-adj < 0.001) between m^6^Acluster were identified to explore the underlying biological functions (**Supplementary Table S1**). GO analysis revealed that the DEGs had a significant enrichment in a number of cell cycle biological processes, including mitotic nuclear division, mitotic sister chromatid segregation, nuclear chromosome segregation, regulation of chromosome segregation, and nuclear division (**Figure 2D**, **Supplementary Table S2**). KEGG analysis indicated that cell cycle and metabolic pathways such as DNA replication, cellular senescence, bile secretion, Glycolysis/Gluconeogenesis, biosynthesis of amino acids were significantly enriched, as well as cancer-related pathways such as ECM-receptor interaction and p53 signaling pathway (**Figure 2E**, **Supplementary Table S3**). KEGG pathway annotation results revealed that many cancer-related pathways were identified, including those with functions in the immune and endocrine system, signaling transduction, DNA/RNA replication and repair, cell growth and death, and metabolism (**Figure 2F**). To explore the underlying biological mechanism of distinct m^6^Acluster subtypes, GSEA and GSVA analyses were conducted. The GSEA analysis also prompted that signaling transduction/cell cycle-related pathways were highly activated in m^6^Acluster B while metabolism biological processes were highly activated in m^6^Acluster A (**Figure 2G**, **Supplementary Table S4**). In addition, a direct comparison of hallmark pathway expression using GSVA revealed a strong enrichment of signaling transduction and metabolism in m^6^Acluster B versus A, such as fatty acid and bile acid metabolism, oxidative phosphorylation, IL2-STAT5 signaling, MYC targets, PI3K-AKT-mTOR signaling, E2F targets, and G2M checkpoint (**Figure 2H**, **Supplementary Table S5**). All above results demonstrated that m^6^Acluster subtypes was correlated with dysregulation of signaling transduction and metabolism, which may be implicated in the poor prognosis of TCGA-LIHC patients.

### 3.3 Weighted gene co-expression network construction and selection of feature genes

**Figure 3:**
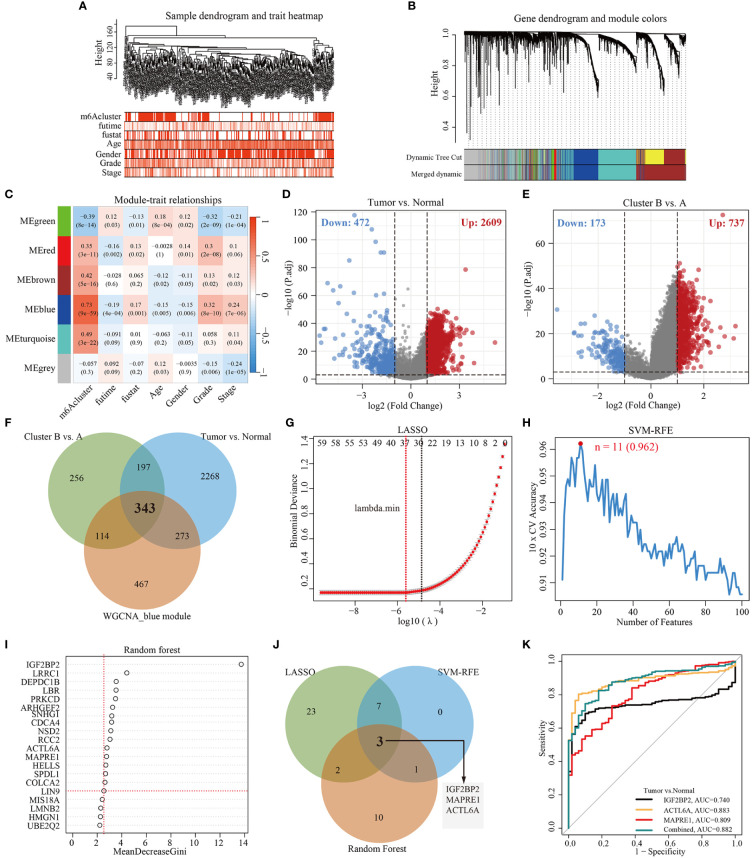
Construction of WGCNA and selection of feature genes. **(A)** Clustering dendrogram of 344 samples with clinical trait heatmap in TCGA-LIHC database. **(B)** Gene clustering dendrograms showing the original and combined modules, various colors represent different modules. **(C)** The relationship of seven traits (including m6Acluster and clinicopathology) and six modules, red and blue represents positive and negative correlations, respectively. Each cell contains the corresponding correlation value and *p*-value. **(D)** Volcano plot of DEGs between tumor and normal tissues. **(E)** Volcano plot of DEGs between cluster B and cluster A. **(F)** Venn diagram demonstrating 343 overlapping genes between the WGCNA blue module gene and the identified DEGs. **(G)** Cross-validation for selecting the optimal tuning parameter log (λ) in LASSO regression algorithm. **(H)** Eleven feature genes were identified by SVM-RFE algorithm with a 10-fold cross-validation accuracy of 0.962. **(I)** Gene importance scores in RF model. MeanDecreaseGini score greater than 2.5 was selected for the inclusion threshold of feature genes. **(J)** Venn diagram demonstrating three diagnostic markers shared by three algorithms (LASSO, SVM-RFE, and Random Forest). **(K)** Performance of three biomarker genes in discriminating tumor from normal controls based on TCGA-LIHC database.

To identify m^6^Acluster-related modules, WGCNA was constructed based on the expression profiles of TCGA-LIHC and clinical trait. Here, we selected the top 5000 genes with the lowest median absolute deviation (MAD) to build a co-expression network. A dendrogram of 344 samples with complete clinical information was clustered using the average linkage method and Pearson’s correlation method, and no discrete samples were found (**Figure 3A**). Next, the power value of *β* = 7 (scale-free topology fitting index *R^2^* = 0.85) was selected as the soft threshold to construct a scale-free network with high average connectivity (**Supplementary Figures S3A, S3B**). After merging the similar modules using two settings: clustering height = 0.3 and min module size = 40, six modules were identified for subsequent analysis (**Figure 3B**, **Supplementary Figure S3C**). Through the transcription correlation study within modules, there was no substantial linkage between modules (**Supplementary Figure S3D**). The relevance between ME (Module Eigengene) and clinical features (m^6^Acluster, fu-time, fu-stat, age, gender, grade, and stage) was evaluated based on module-trait relationships (MTRs). The module-trait relationship results indicated that the MEblue (r = 0.73, *P* = 9e-59), the MEbrown (r = 0.42, *P* = 5e-16), the MEred (r = 0.35, *P* = 3e-11), the MEgreen (r = -0.39, *P* = 8e-14) are significantly associated with m^6^Acluster (**Figure 3C**). Moreover, the MEblue and MEgreen were significantly related to other clinical features, and the two modules showed an inverse correlation trend. Considering the high correlation with m^6^Acluster, we selected the MEblue module as the target module for the subsequent study. The scatterplot of GS versus MM indicated that significant correlation existed in the module membership (MM) and gene significance (GS) of the MEblue (cor = 0.48, *P* = 1.6e-58) module (**Supplementary Figure S3E**).

Here, the differentially expressed genes (DEGs) (|log2FoldChange| > 1, *P*-adj < 0.001) between different cohorts were illustrated by the volcano plot. As shown in **Figures 3D** and **3E**, a total of 3081 DEGs (2609 up-regulation and 472 down-regulation) were identified between tumor and tumor-adjacent tissues, and 910 DEGs (737 up-regulation and 173 down-regulation) between m^6^Acluster A and cluster B. Then, 343 overlapping genes were obtained by intersecting the blue module genes and the differential genes using a Venn diagram (**Figure 3F**). To identify key feature genes, the 343 candidate genes were submitted into LASSO regression algorithm, SVM-RFE algorithm, and RF model. LASSO regression analyses with a 10-fold cross-validation identified thirty-five gene signatures (**Figure 3G**). An eleven-gene signature was identified by SVM-RFE algorithm with a 10-fold cross-validation accuracy of 0.962 (**Figure 3H**). The RF model algorithm sorted sixteen gene signatures with MeanDecreaseGini scores greater than 2.5 (**Figure 3I**). To obtain a robust feature gene for m^6^Acluster, we intersected the genes screened out by the above three algorithms and identified three key feature genes: *IGF2BP2*, *MAPRE1*, and *ACTL6A*, as shown in **Figure 3J**. The ROC curves of *IGF2BP2*, *MAPRE1*, and *ACTL6A* revealed the probability of them as valuable biological markers with AUCs higher then 0.7 (**Figure 3K**), indicating that the three diagnostic markers had a higher diagnostic value. Furthermore, our PCR results demonstrated that the expression levels of *ACTL6A*, *MAPRE1*, and *IGF2BP2* were upregulated in HCC tissues compared to adjacent tissues (*p* < 0.01, as shown in **Supplementary Figure S4**).

### 3.4 Construction and evaluation of m^6^Arisk scoring model

**Figure 4:**
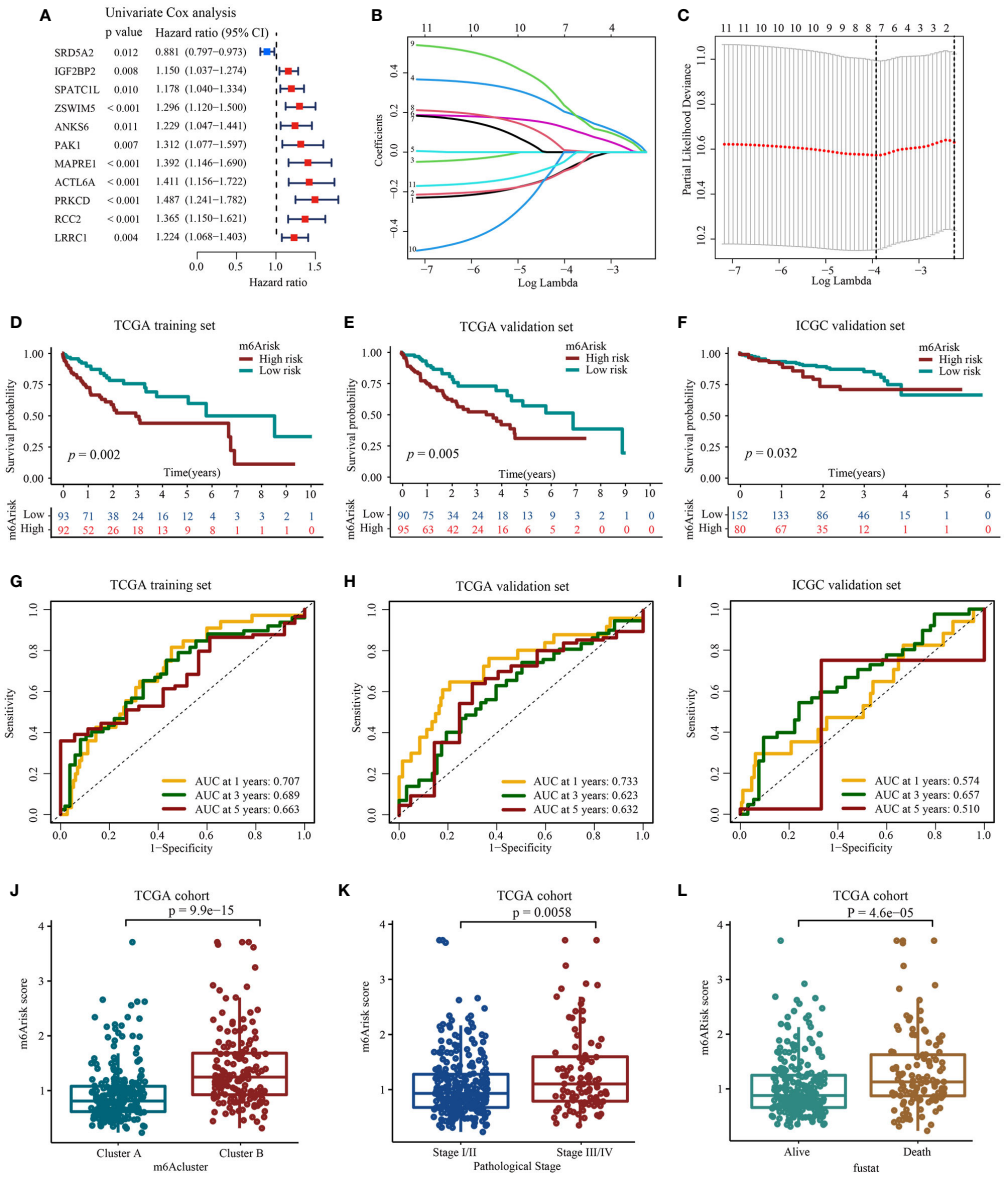
Construction and evaluation of prognostic signature using m^6^A-related candidate genes. **(A)** Univariate Cox regression analysis. **(B, C)** LASSO regression analysis and optimal parameter (lambda) selection of the eleven prognostic genes by using 10-fold cross-validation. Dotted vertical lines represents the optimal values selected by the minimum criteria (right) and the 1- standard error (SE) of the minimum criteria (left). **(D)** Development of m^6^Arisk model in TCGA-LIHC training set **(E)** Validation of the m^6^Arisk model in TCGA-LIHC internal validation set. **(F)** Validation of the m^6^Arisk model in external independent validation sets: ICGC-LIRI-JP. **(G–I)** The predictive accuracy of m^6^Arisk model for survival. **(J)** Differences in m^6^Arisk score between two distinct m6Acluster subtypes. **(K)** Differences in m^6^Arisk score between HCC patients with AJCC stages III–IV and stages I-II. AJCC, American Joint Committee on Cancer. **(L)** Differences in m^6^Arisk score between HCC patients who had deceased and HCC patients who were alive.

To explore potentially valuable prognostic genes more broadly, we included overlapping genes that appeared in any two algorithms for subsequent analysis. Overall, 11 out of thirteen genes were found to affect prognosis based on univariate Cox analysis (**Figure 4A**, **Supplementary Table S6**). Next, we performed LASSO and multivariate Cox regression analysis for eleven prognostic genes to further select optimum prognostic signature. Followed by LASSO analysis, seven best candidate DEGs (*SRD5A2*, *IGF2BP2*, *ZSWIM5*, *PAK1*, *ACTL6A*, *PRKCD*, *LRRC1*) were retained according to the minimum partial likelihood deviance (**Figures 4B, C**). Subsequently, the seven candidate DEGs underwent multivariate Cox analysis, resulting in the retention of four genes (*SRD5A2*, *IGF2BP2*, *ZSWIM5*, *PRKCD*) according to the Akaike information criterion (AIC) value. Consequently, the m^6^Arisk score model was developed according to RNA-expression profiles using the following formula: Risk score = (−0.1430* expression of *SRD5A2*) + (0.2223*expression of *IGF2BP2*) + (0.2784* expression of *ZSWIM5*) + (0.4081* expression of *PRKCD*). As shown in **Supplementary Figure S4**, HCC tissues exhibited decreased *SRD5A2* expression levels (*p* < 0.01), while *ZSWIM5, PRKCD*, and *IGF2BP2* expression levels (*p* < 0.01) were upregulated compared to adjacent tissues.

After the construction of m^6^Arisk score model, we performed evaluation and validation analysis of the risk model. In the TCGA-LIHC training dataset, 185 patients were divided into high m^6^Arisk score group (n=92) and low m^6^Arisk score group (n=93) using the median m^6^Arisk score as the risk cutoff. As shown in **Figures 4D, G**, individuals with elevated m^6^Arisk scores experienced notably shorter overall survival (OS) times compared to those with lower m^6^Arisk scores. The area under the curve (AUC) values for the m^6^Arisk scoring model were 0.707, 0.689, and 0.663 for the 1-year, 3-year, and 5-year OS periods, respectively. The predictive accuracy of the m^6^Arisk scoring model was well validated in TCGA-LIHC internal validation cohort, with AUC values of 0.733, 0.623, and 0.632 for 1-, 3-, and 5-year OS, respectively (**Figures 4E, H**). In addition, we further verified the predictive capacity of the m^6^Arisk scoring model in external ICGC-LIRI-JP cohort (**Figures 4F, I**). As shown in **Figure 4J**, a significant difference in the distribution of m^6^Arisk scores was observed between m^6^Acluster A and B. The risk scores of the patients in m^6^Acluster B were substantially higher than those of the patients in m^6^Acluster A. We also determined the relationship between m^6^Arisk score and clinicopathological features of HCC patients. HCC patients diagnosed with AJCC stages III–IV had significantly higher m^6^Arisk scores than those diagnosed with stage I-II (**Figure 4K**). Similarly, the m^6^Arisk score of patients who died was significantly higher than that of patients who survived (**Figure 4L**). These results indicate that the m^6^Arisk scoring model may serve as a powerful indicator for the prognosis of liver cancer patients.

### 3.5 The m^6^Arisk score significantly correlates with tumor immune phenotypes of HCC

**Figure 5:**
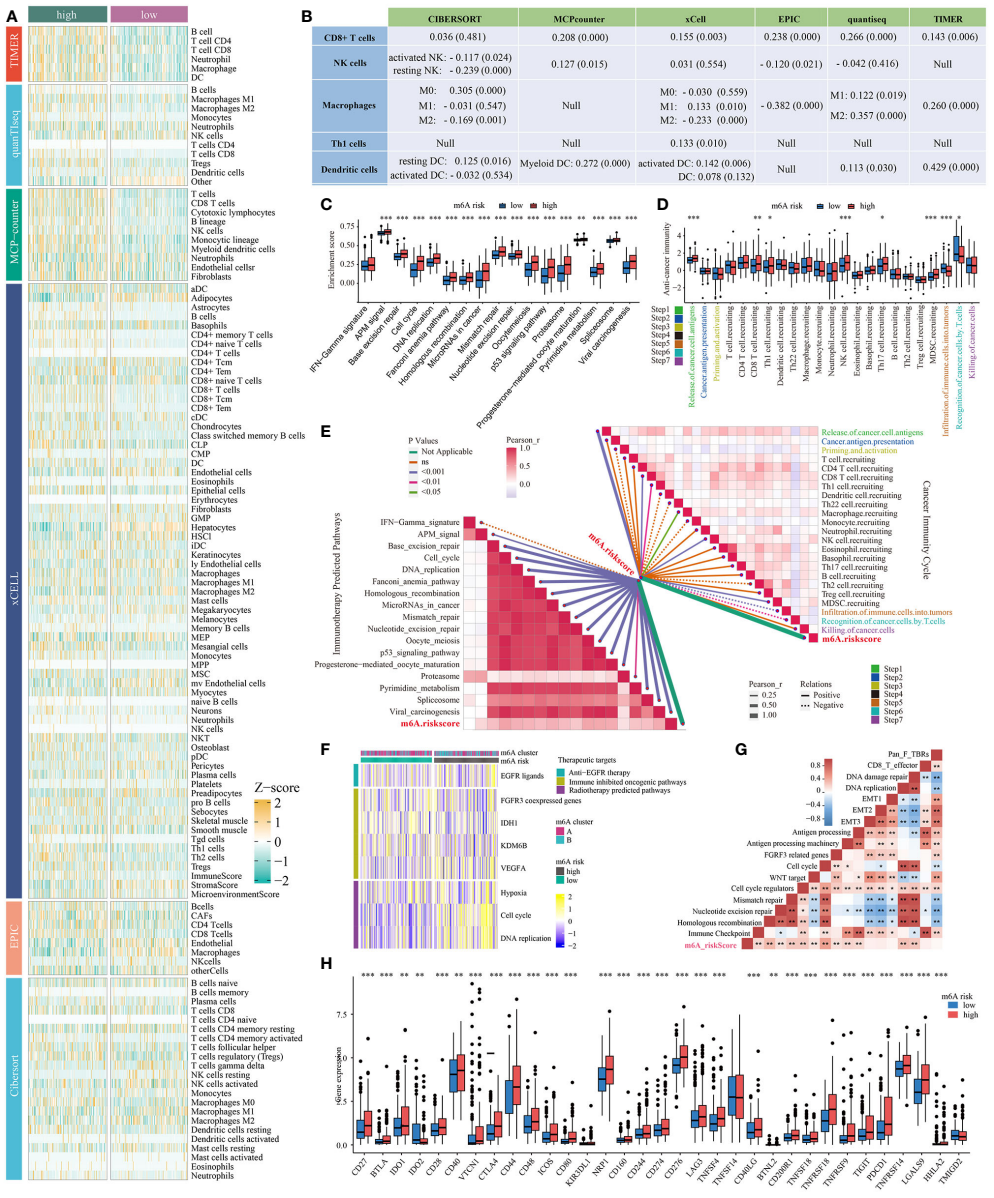
Correlation between the m^6^Arisk score and immune phenotypes. **(A)** Six independent algorithms including CIBERSORT, MCP-counter, xCell, EPIC, quantiseq, and TIMER, further verified the stability and robustness of the ssGSEA results. **(B)** Correlation between m6Arisk score and the infiltration levels of five types of TIICs (CD8+ T cells, NK cells, macrophages, Th1 cells, and dendritic cells). **(C)** Differences in the enrichment scores of immunotherapy-predicted pathways between the two m6Arisk groups in TCGA-LIHC cohort. The enrichment scores were calculated using ssGSEA algorithms. **(D)** Differences in the various steps of the cancer immunity cycle between the two m6Arisk groups in TCGA-LIHC cohort. **(E)** Pearson’s correlation analysis of the m^6^Arisk score with cancer immunity cycle activity (top right) and immunotherapy-predicted pathways (bottom left) based on TCGA-LIHC cohort. The color of the line represents the size of the *P* value, and the thickness of the line represents the size of the r value. The solid and dotted lines represent positive and negative correlations, respectively. **(F)** Correlations between m^6^Arisk scores and the enrichment scores of several therapeutic signatures such as targeted therapy and radiotherapy. **(G)** Correlations between m^6^Arisk scores and the known biological gene signatures using Spearman analysis. The color presented the Spearman correlation coefficient. **(H)** Difference analysis of immune checkpoints effect genes between high- and low-m^6^Arisk groups in TCGA-LIHC cohort. * *P* < 0.05; ** *P* < 0.01; *** *P* < 0.001. ns, No significance.

Here, we investigated the existence of immune heterogeneity in different m^6^Arisk score groups, and the association between the m^6^Arisk score and various immune characteristics (expression of immunomodulator and TIIC effector genes, immunotherapy-related characteristics, and immune checkpoints). As shown in **Figure 5A**, **Supplementary Table S7**, we first investigated the infiltration level of Tumor infiltrates immune cells (TIICs) using six independent algorithms. The result indicated that the m^6^Arisk score was positively correlated with the infiltration level of CD8+ T cells, dendritic cells, and macrophages under different algorithms (**Figure 5B**; **Supplementary Table S8**). As expected, m^6^Arisk score was also found to be positively correlated with the effector genes of these TIICs (**Supplementary Figures S5A, S5B**). We also analyzed the correlations between m^6^Arisk score and the immunotherapy predicted pathways signatures (**Supplementary Tables S9–S11**). As shown in **Figures 5C, E**, the m^6^Arisk score was positively correlated with a majority of the immunotherapy predicted-related pathways, including IFN-Gamma signature, base-excision repair, cell cycle, Fanconi anemia pathway, p53 signaling pathway, MicroRNAs in cancer, proteasome, and pyrimidine metabolism.

In addition, the activities of a portion of the cancer immunity cycle were also found to be upregulated in the high-m^6^Arisk score group, including the release of cancer cell antigens (Step 1) and trafficking of immune cells to tumors (Step 4, mainly those that exert antitumor immunity), such as CD8 T cell recruiting, NK cell recruiting, and MDSC recruiting (**Figure 5D**, **Supplementary Table S12**). The activities of the cancer immunity cycle are a direct comprehensive performance of the functions of the chemokine system and other immunomodulators (25, 27). The elevated activity of these steps might increase the infiltration levels of effector TIICs in the TME. Interestingly, the activity of infiltration of immune cells to tumors (Step 5) and recognition of cancer cells by T cells (Step 6) was upregulated in the low-m^6^Arisk score group. Moreover, the correlation analysis indicated that m^6^Arisk score demonstrated a predominantly positive correlation with the critical steps of cancer-immunity cycle (Step 1 and Step 4) and the enrichment scores of immunotherapy-predicted pathways gene signatures, including the interferon-γ signature, base-excision repair, cell cycle, DNA replication, homologous recombination, the p53 signaling pathway, and others (**Figure 5E**, **Supplementary Table S11**).

In addition, the enrichment scores for several immunosuppressive oncogenic pathways (such as radiotherapy-predicted pathways and EGFR ligands) were significantly higher in the high-m6Arisk group (**Figure 5F**; **Supplementary Tables S13**, **S14**). Previous studies have found that inhibiting these oncogenic pathways promoted the formation of an inflamed tumor microenvironment (TME), thereby reactivating cancer immunity. We also examined the relationship between known biological signatures and the m^6^Arisk score through Spearman analysis. A heatmap of the correlation matrix demonstrated that the m^6^Arisk score was markedly positively correlated with the immune activation process and DNA repair signatures (**Figure 5G**, **Supplementary Tables S15**, **S16**). Consistently, a significant proportion of immune checkpoint genes were observed to be highly expressed in the high-risk score group within this study, such as CD27, CD28, CD40, CTLA4, CD44, CD48, NRP1, CD276, LAG3, TNFSF4, PDCD1(PD-1), and TIGIT (**Figure 5H**). Similarly, another heatmap was drawn to show the mRNA expression profiles of immunomodulator genes including chemokine, immune inhibitor, immune stimulator, MHC, and receptor in two m^6^Arisk score groups (**Supplementary Figure S5C**). The m^6^Arisk score positively correlated with the mRNA expression profiles of immunomodulator genes. Most MHC molecules were upregulated in the high-m^6^Arisk group, suggesting that antigen presentation and processing capacity were upregulated in the high-m^6^Arisk group. The chemokines, including *CCL4*, *CCL5*, *CCL8*, *CCL20*, *CCL26*, *CXCL1*, *CXCL3*, *CXCL5*, *CXCL9*, *CXCL11*, *CXCL16*, and paired receptors including *CCR1*, *CCR5*, *CXCR3*, *CXCR4*, and *CXCR6*, were positively correlated with m^6^Arisk score. These chemokines and receptors promote the recruitment of effector TIICs such as CD8+ T cells and antigen-presenting cells. However, given the complex and diverse functions of the chemokine system, although the relationship between m6Arisk score and individual chemokines is not sufficient to clarify the overall immune effect of m6Arisk in TME, it also reflects that the high score of m6Arisk is closely related to the development of inflammatory TME to some extent.

### 3.6 Genomic alterations between different m^6^Arisk score groups

**Figure 6:**
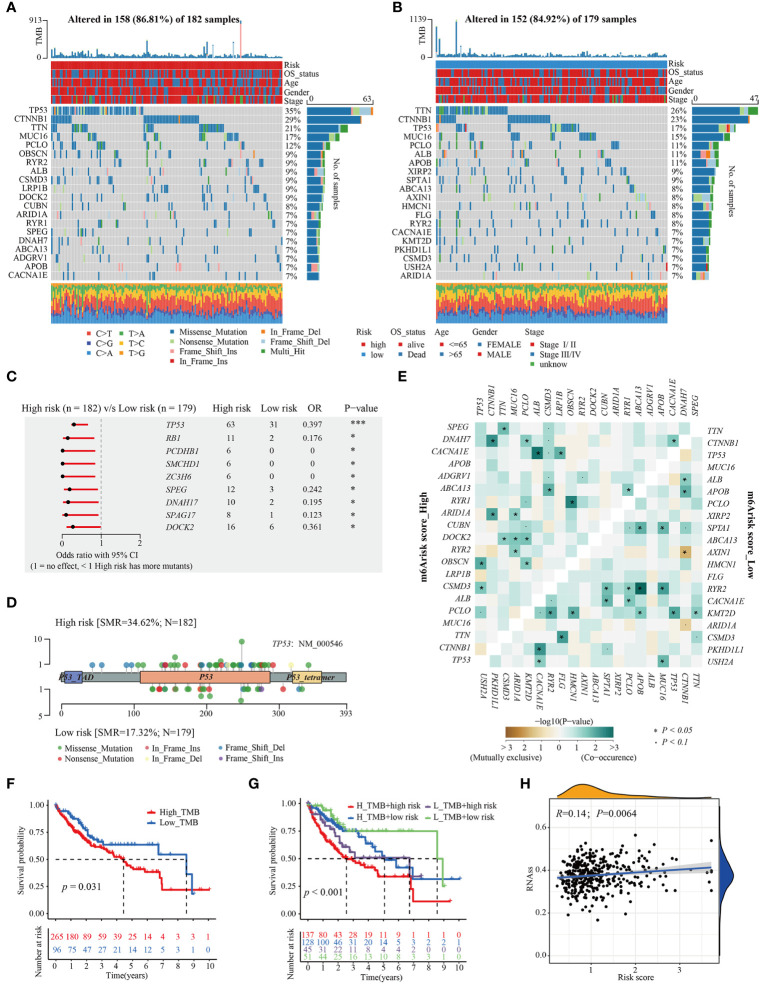
Distinctive genomic mutation patterns between the m6Arisk score groups. **(A, B)** Waterfall plots depicting the somatic mutation landscapes of the top 20 most frequently mutated genes in the high- and low-m^6^Arisk score groups. **(C)** Forest plot displaying the common driver genes mutating significantly differentially in the high- and low- m6Arisk score groups. **(D)** Lollipop diagram visualizing the differential mutation site for TP53 between the two distinct m6Arisk score groups. **(E)** The mutual exclusivity and co-occurrence of mutations in the most frequently mutated genes of the high- and low-m6Arisk score groups. **(F)** Kaplan-Meier curves of TMB in the high- and low-m^6^Arisk score groups. **(G)** Kaplan-Meier curves for HCC patients in the whole TCGA-LIHC cohort stratified by both TMB and m^6^Arisk score. TMB, tumor mutation burden. **(H)** Relationships between m^6^Arisk score and cancer stem cell (CSC) index. ***P < 0.001, *P < 0.05.

To give a hint of m6Arisk-related mechanisms for OS classification of HCC from genomic layer, available somatic mutations of the TCGA-LIHC dataset were acquired, and the distribution differences in the high- and low-m6Arisk groups were analyzed by the package “maftools”. **Figures 6A, B** showed the top 20 genes with the highest mutation frequencies in the two m^6^Arisk-score groups. The summary of the mutation information, along with statistical calculations, is presented in **Supplementary Figures S6A, S6B**. *TP53* (35%) and *TNN* (26%) were the most frequently mutated genes in the high- and low-m^6^Arisk patients, respectively, with *TP53* having the highest frequency. The Forest plot (**Figure 6C**) illustrates genes with significant differences in mutation frequency between the two m^6^Arisk score groups, including *TP53*, *RB1*, *PCDHB1*, *SMCHD1*, *ZC3H6*, *SPEG*, *DNAH17*, *SPAG17*, and *DOCK2*. As *TP53* was the most frequently mutated gene, a lollipop diagram (**Figure 6D**) was created to illustrate the specific mutation sites of *TP53*, with a higher number of missense mutations observed in the high-m^6^Arisk group. Furthermore, the associations of exclusivity and co-occurrence across mutated genes from the high- and low-m^6^Arisk score groups are shown in **Figure 6E**, with green representing co-occurrence and brown representing mutual exclusion. Here, the tumor mutation burden (TMB) quantification results demonstrated an elevated level in the high-m6Arisk group, although in a non-significant mode (**Supplementary Figure S6C**), and HCC patients with a lower TMB score presented a better overall survival (OS) (**Figure 6F**). This finding suggests the presence of heterogeneity and complexity among cancer patients, which is consistent with existing literature reports (44). To further investigate, we categorized all HCC patients into four subgroups based on TMB and m^6^Arisk score: high-TMB and high-m^6^Arisk, low-TMB and high-m^6^Arisk, high-TMB and low-m^6^Arisk, and low-TMB and low-m^6^Arisk. Survival curves were plotted for each subgroup, and it was observed that the high-TMB and high-m^6^Arisk score group exhibited the worst prognosis among them (**Figure 6G**). We then assessed the potential correlation between the m^6^Arisk score and the cancer stem cell (CSC) index in HCC. As shown in **Figure 6H**, a positive linear correlation between the m^6^Arisk score and CSC index was observed (R = 0.14, *P* < 0.01). The results suggest that HCC cells with a higher m^6^Arisk score may have more pronounced stem cell properties and a lower degree of cell differentiation.

### 3.7 The m^6^Arisk score predicts therapeutic responses in HCC

**Figure 7:**
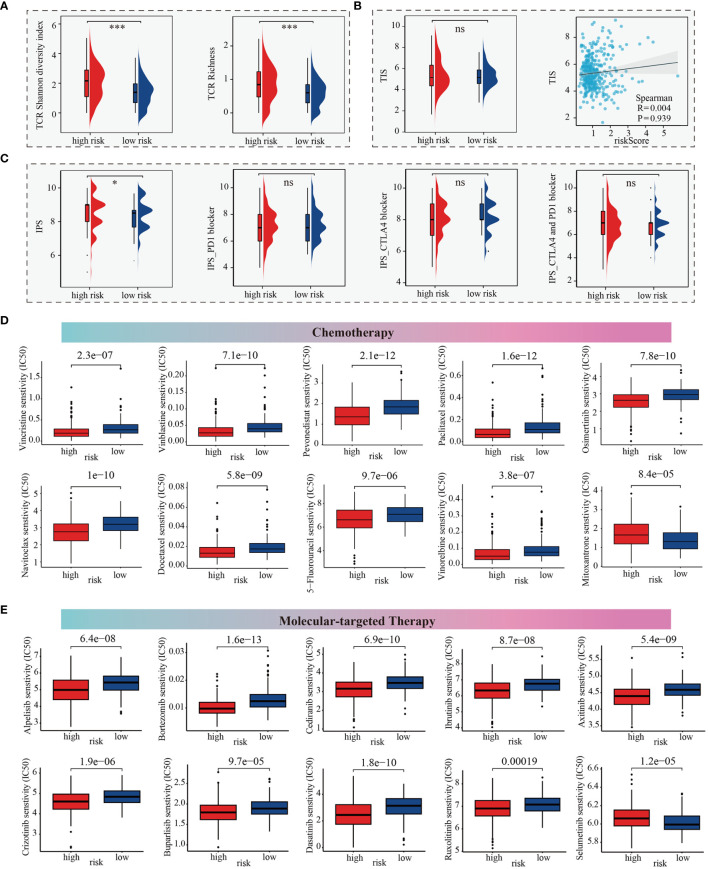
m^6^Arisk score based prediction of treatment response. **(A)** TCR repertoire analysis illustrating significantly higher levels of TCR richness and diversity in the high-m^6^Arisk score group based on the TCGA-LIHC cohort. **(B)** Comparison of TIS between the two distinct m^6^Arisk score groups based on the TCGA-LIHC cohort. **(C)** IPS comparison of the high- and low- m^6^Arisk score groups based on the TCGA-LIHC cohort. **(D)** Boxplots depicting differential sensitivities of common chemotherapeutic drugs between the two distinct m^6^Arisk score groups. **(E)** Differential sensitivities of common molecular-targeted therapeutic drugs between the distinct m^6^Arisk score groups. *, P <0.05; ***, P <0.001; ns, No significance.

Here, we firstly estimated the T cell receptor (TCR) repertoire for HCC patients and HCC patients (TCGA-LIHC cohorts) in the high-m^6^Arisk score group exhibited a significantly higher TCR richness and diversity, indicating that they possessed greater tumor immune potential (**Figure 7A**). Besides, the Tumor Inflammation Signature (TIS), an 18-gene index that measures adaptive immune resistance within tumors, was utilized to evaluate the immune potential of the two risk groups. As shown in **Figure 7B**, patients in the two m^6^Arisk score groups exhibited a non-significant TIS score, indicating no significant difference in anti-tumor immune potential. Imunophenoscore (IPS) is a recognized indicator of patients’ response to immunotherapy, and no significant differences were observed between the two m^6^Arisk score groups, suggesting no difference in response to immune checkpoint blockade (ICB) between the two groups (**Figure 7C**). These results suggest that the m6Arisk score may not help identify effective anti-tumor immunotherapy precision medicine therapies.

We subsequently investigated whether the m^6^Arisk score could accurately guide precision treatments by assessing the differences in anticancer drug sensitivity between the two m^6^Arisk score subgroups, aiming to identify potential individualized therapy modalities for LIHC patients. The IC50 values demonstrated that LIHC patients with a lower m^6^Arisk score exhibited a higher sensitivity to common chemotherapeutic drugs, including vincristine, vinblastine, pevonedistat, paclitaxel, osimertinib, navitoclax, docetaxel, vinorelbine, and 5-fluorouracil (**Figure 7D**). Additionally, LIHC patients with lower m^6^Arisk score also showed higher sensitivity to several targeted drugs, such as alpelisib, bortezomib, cediranib, ibrutinib, axitinib, crizotinib, buparlisib, dasatinib, and ruxolitinib (**Figure 7E**). In contrast, patients with a high m^6^Arisk score exhibited relatively high sensitivity to the chemotherapy drug mitoxantrone (**Figure 7D**) and the targeted drug selumetinib (**Figure 7E**). These results demonstrate that the m^6^Arisk score may contribute to identifying effective antitumor agents and precision medicine therapies for LIHC treatment.

### 3.8 Construction and validation of a nomogram

**Figure 8:**
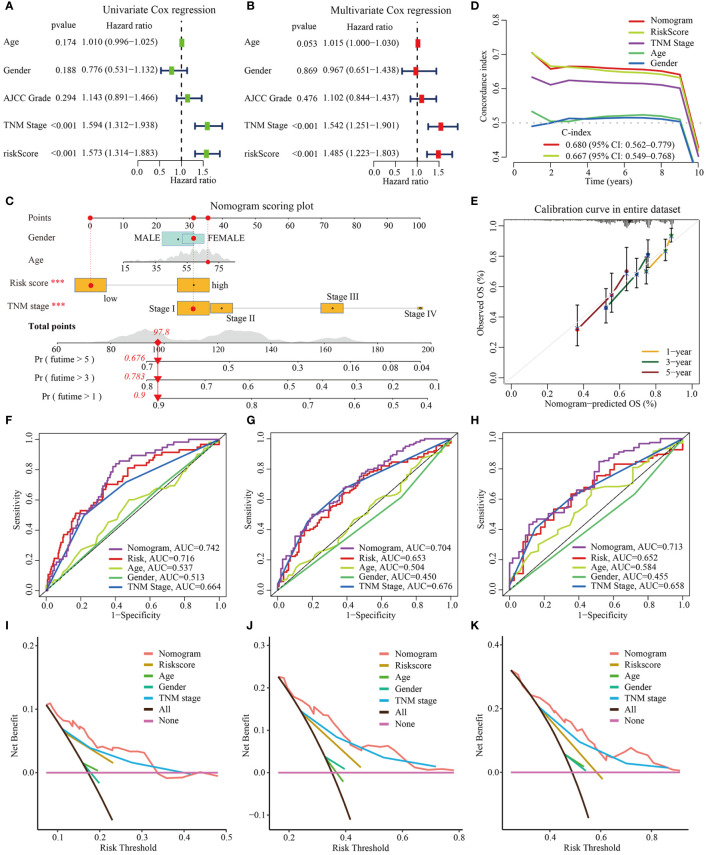
Construction and validation of nomogram based on TCGA-LIHC dataset. **(A, B)** Univariate and multivariate Cox regression analysis for m6Arisk score, respectively. **(C)** The established nomogram for predicting the 1-, 3-, and 5-year OS of HCC patients. The red arrow signifies an example to visualize the assessment of risk for 1-, 3-, and 5-year OS. **(D)** C-indexes for the generated nomogram and single variables in predicting OS of HCC patients. The C-index was estimated by truncating the follow-up time to 1 to 10 years and plotting it on the X-axis as the truncation year. **(E)** Calibration curves of the nomogram in terms of the agreement between predicted and observed outcomes. **(F–H)** The ROC curves of the nomograms and clinical characteristics for predicting 1-year, 3-year, and 5-year OS in HCC patients. (**I–K)** The DCA curves of the nomograms and clinical characteristics for predicting 1-year, 3-year, and 5-year OS in HCC patients. OS, overall survival; DCA, decision curve analysis; ROC, receiver operating curve.

To assess whether the m^6^Arisk scores predicting model was an independent predictor in HCC (TCGA-LIHC cohorts), univariate and multivariate Cox regression analyses were conducted. As shown in **Figures 8A, B**, the HR of m^6^Arisk scores in univariate and multivariate analysis was 1.573 (95%CI: 1.314-1.883; *p*<0.001) and 1.485 (95%CI: 1.223-1.803; *p*<0.001), suggesting that m^6^Arisk scores could be used as an independent prognostic indicator compared with the other clinical features (age, gender, AJCC stage, and TNM stage). To facilitate the clinical feasibility of the m^6^Arisk score, a nomogram was constructed by integrating the m^6^Arisk score and clinicopathological features to predict overall survival (OS) at 1-, 3-, and 5- years. As shown in **Figure 8C**, the predictors included the m^6^Arisk score and TNM stage, which had the greatest influence on OS. We subsequently validated the predictive capability and accuracy of this nomogram by concordance index (C-index), calibration curve, and decision curve analysis (DCA). The C-index of the nomogram was 0.680 (95% CI: 0.562–0.779) in the TCGA-LIHC cohort (**Figure 8D**) and 0.733 (95% CI: 0.553–0.859) in external validation cohort (**Supplementary Figure S7A**), indicating that the nomogram had a relatively good discriminatory power. Similarly, the calibration plots show an ideal consistency between the actual observations and the nomogram predictions of the 1-, 3-, and 5-year OS in both the TCGA-LIHC cohort and external validation cohort (**Figure 8E**, **Supplementary Figure S7B**). The ROC analysis revealed that the AUC values of the constructed nomogram for predicting 1-, 3-, and 5-year OS were 0.742, 0.704, and 0.713, respectively, further demonstrating the predictive capability of the nomogram (**Figures 8F–H**). As showed in **Figures 8I–K**, nomogram incorporating the m^6^Arisk model yielded a relatively better net benefits than other clinical traits in predicting 1-, 3-, and 5-year OS for HCC patients in the TCGA-LIHC cohort, suggesting that the nomogram had a relatively good prognostic accuracy and clinical applicability. The ROC and decision curve (DCA) analysis indicated that the proposed nomogram had a similar performance in the ICGC-LIRI-JP cohort (**Supplementary Figures S7C–S7H**).

## 4 Discussion

Hepatocellular carcinoma (HCC) remains a major health challenge with a growing incidence worldwide today, characterized by high recurrence rates and heterogeneity (45). The existing prognostic staging system still has some limitations in evaluating clinical prognosis and individual treatment for HCC patients. How to control its progression and improve the survival rate of patients remains an urgent issue to be solved in the current treatment of liver cancer. Accumulating evidence demonstrates that hepatocellular carcinogenesis is regulated by complex genetic and epigenetic mechanisms, and influenced by immune cell infiltration and the tumor microenvironment (46–49). A study using whole-genome and -exome sequencing analysis has shown that epigenetic regulation is the most unusual differential modifier in HCC. As the most predominant epigenetic modification, RNA methylation modification plays an indispensable and pleiotropic biological role in malignant transformation and cancer progression. N^6^-methyladenosine modification affects gene expression by regulating RNA processing, decay, and translation, and abnormal expression of the m^6^A methylase complexes is strongly associated with various human cancers (8, 50–52), including HCC.

Recent studies have shown the impact of m^6^A RNA modification on various inflammatory development of cancer. Inflammation predisposes patients to cancer, especially affecting the composition of the tumor microenvironment and the plasticity of tumor cells, including surrounding stromal and inflammatory cells (53). m^6^A dysregulation may lead to aberrant expression of oncogenic or the tumor-suppressive genes, contributing to HCC initiation and progression. m^6^A dysregulation may also contribute to epigenetic alterations in HCC cancer cells, and may affect cancer stem cell potential, thereby impacting tumor growth and therapy resistance (54). Besides, another study indicated that the construction of polygenic risk prediction model based on m^6^A related genes has good clinical predictive ability and accuracy in predicting the survival and prognosis of glioma patients, and is an independent risk factor for glioma. These results suggest that the construction of polygenic risk prediction models based on m^6^A associated genes has different potential in the stratification of cancer prognosis and the development of new treatment strategies. Thus, comprehensively investigating m^6^A modification in HCC and its biological roles may facilitate improved prognostic predictions and individual precise treatment modalities for HCC. In this study, we identified two distinct m^6^A modification patterns in HCC, each being associated with immunological properties, therapeutic response, and prognoses. Finally, we further developed an m^6^Arisk score model to quantify the m^6^Arisk subtype in HCC patients and independently validated this model using the ICGC-LIRI-JP cohorts.

In this study, we found that these m^6^A regulatory genes present a tight and highly interconnected molecular interaction network, which are mainly involved in mRNA stability, mRNA transport, and mRNA metabolism. Analysis of copy number alterations (CNA) and expression profiles revealed a significant abnormal imbalance in the expression levels of m^6^A writers, readers, and erasers between tumor and normal tissues. In theory, these imbalances could lead to aberrant m^6^A modification patterns, ultimately contributing to HCC formation and progression. Furthermore, based on the expression profiles of 23 m^6^A regulators, we identified two independent m^6^A modification patterns in the TCGA-LIHC cohort using the consensus unsupervised clustering algorithm. Subsequent survival analysis revealed significantly worse prognoses for HCC patients in m^6^Acluster B compared to those in m^6^Acluster A. Additionally, we observed that cluster-specific DEGs were also associated with cell cycle and metabolic pathways, as well as cancer-related pathways, such as ECM-receptor interaction and p53 signaling pathway. These findings provide further insights into the potential biological mechanisms underlying the distinct m^6^A modification patterns and their implications in HCC development and progression.

Moreover, we identified modules significantly correlated with clinical features and m^6^Acluster subtypes in the subsequent WGCNA based on TCGA-LIHC cohort. To screen potential prognostic biomarkers, we performed three different algorithms (LASSO, SVM-RFE and RF) on the above overlapping 343 DEGs. We also developed a robust m^6^Arisk score model based on the expression of four m^6^A-related genes. Our results indicated that the m^6^Arisk score performed well in predicting the prognoses of HCC patients. Particularly, a high m^6^risk score was significantly associated with poorer clinical outcomes and lower drug sensitivity. In clinical practice, the TNM stage is a conventional reference for evaluating clinical outcomes and treatment decisions. Surprisingly, multi-Cox regression analysis further validated the superiority of the established m^6^Arisk score model in predicting OS in HCC patients, independent of other clinical features such as age, gender, and TMN stage. Finally, by integrating the m^6^Arisk score and clinical features, we developed a quantitative nomogram that enhances the clinical operability of m^6^Arisk score. The prognostic model can be used for stratifying the prognosis of HCC patients and provides new ideas for targeted therapies. Moreover, the patients in the high- and low-m^6^Arisk score groups presented distinct clinicopathological features, mutation patterns, immune cell infiltration and immune checkpoint characteristics.

With in-depth research on tumor immunology, immunotherapy has emerged as a promising strategy for tumor treatment. Immune checkpoint blockade (ICB) is currently the most successful and common immunotherapy strategy (55, 56). Currently, PD-1/PD-L1 monoclonal antibodies have become important targeted therapeutic drugs for a variety of tumor immunotherapy. Thus, the therapy immunotherapy strategies targeting m6A methylation provide direction for a direction for improving the therapeutic efficacy of immune checkpoint inhibits. Previous studies have shown that epigenetic-based targeted therapies and immunotherapies work better in clinical tries (57). A study on HCC stem cells found that knockdown AMD1 leaded decreased FTO to regulate m6A methylation levels, which reduced the resistance of HCC cells to sorafenib. They also verified the specific inhibitor of AMD1 may be an effective alternative agent for the treatment of HCC in combination with sorafenib (58). In a similar study of lung cancer, targeting the m6A methylation regulatory enzyme could inhibit cancer cell growth or increase the sensitivity of anti-cancer drugs (59). In glioblastoma, reversing temozolomide resistance conferred by m6A methylation could aid in the development of new therapeutic interventions (60). Another study showed that targeted m6A therapy mediated by knockdown of ALKBH5 expression participated in and promoted angiogenesis, which may also play a role in HCC, providing a new avenue for combined immunotherapy (61). Although clinical immunotherapy (such as anti-PD-1, anti-PD-L1, and anti-CTLA-4) for HCC has been widely used for HCC worldwide (62, 63), only a minority of patients benefited from immunotherapy. Therefore, there is an urgent need for more effective biomarkers to assess whether patients with HCC benefit from tumor immunotherapy. In this study, our findings indicated that high-m^6^Arisk group appeared to coexist with high expression levels of common immune checkpoint molecules (such as CTLA-4, PDCD1(PD-1), and TIGIT), indirectly suggesting that m^6^Arisk score may be a better predictor of immunotherapy in HCC patients. The upregulation of immune checkpoints such as PD-L1/PD-1 is a critical characteristic of an inflamed TME, which is driven by pre-infiltrating tumor infiltrating immune cells (TIICs) (64). These immune checkpoints suppress pre-existing cancer immunity to avoid an excessive immune response, but also lead to immune evasion. Here, the expression of immune checkpoints (such as CTLA-4, PDCD1(PD-1), and TIGIT) was significantly upregulated in the high-m^6^Arisk group, which might be attributed to the upregulation of pre-existing TIICs. These results suggested that the HCC patients with high-m^6^Arisk score were more sensitive to immune checkpoint blockade (ICB). However, in this study, immunophenotypic scores (IPS) showed no significant difference in response to ICB between the two m^6^Arisk score groups. This might be due to the complexity and multiple functions of the TME system, the relationship between m^6^Arisk and individual immune checkpoints was insufficient to clarify the overall immunological effect of m^6^Arisk in TME.

Moreover, we also observed a positive correlation of m^6^Arisk score with the infiltration level of CD8^+^ T cells under different algorithms. A growing number of studies have evaluated the contribution of cytotoxic cells, especially CD8^+^ T cells. The cancer immunity cycle represents the immune response of our body to cancer. The activities of the cancer immunity cycle are a direct reflection of the final effect of complex immunomodulatory interactions in tumor microenvironment (TME). In this study, we noted that m^6^Arisk score presented a positive correlation with the activities of a portion of the cancer immunity cycle. For example, the release of cancer cell antigens (Step 1) and trafficking of immune cells to tumors (Step 4, mainly those that exert antitumor immunity), such as CD8 T cell recruiting, NK cell recruiting, and MDSC recruiting, was significantly upregulated in the high-m^6^Arisk group. Consequently, the infiltration levels of several effector TIICs, such as CD8+ T cells, dendritic cells, and macrophages, were also significantly increased in the high-m^6^Arisk group, which had been validated in six different algorithms. Therefore, the high m^6^Arisk-score reflected an inflammatory phenotype in TME. Meanwhile, m^6^Arisk score was positively correlated with the enrichment scores of immunotherapy-predicted pathways.

Besides, our findings further indicated that HCC patients with a high m^6^Arisk score were more sensitive to some common chemotherapy and molecular-targeted drugs, suggesting that the m^6^Arisk score might contribute to guiding personalized treatment for patients. However, the drug mechanisms and their effects on HCC progression need to be further studied. Additionally, we developed a nomogram model by incorporating the m^6^Arisk score and clinicopathological features, and further validated and evaluated the predictive capability and accuracy of this model in external verification cohort. These results suggested that the application of the m^6^Arisk score for the prognostic stratification of HCC has good clinical applicability and clinical net benefit.

Finally, it’s worth noting that despite its intriguing and promising findings, this study has several limitations. First, this study is a retrospective study based on public online databases (TCGA-LIHC and ICGC-LIRI-JP), which may have inherent selection bias. Second, although our results were generalized and robust in validation cohorts, the batch effects from different cohorts should be considered. Third, although we highlighted the predictive power of m^6^Arisk scores for HCC TME status and prognosis, we did not identify the molecular mechanisms involved.

## 5 Conclusion

In our study, our findings reveal the crucial role of m^6^A modification patterns for predicting HCC TME status and prognosis, and highlight the good clinical applicability and net benefit of m^6^Arisk score in terms of prognosis, immunophenotype, and drug therapy in HCC patients.

## Data availability statement

The original contributions presented in the study are included in the article/**Supplementary Materials**, further inquiries can be directed to the corresponding author/s.

## Ethics statement

Twenty-eight pairs of fresh-frozen tissues (HCC tissues and adjacent tissues) were collected from the Zhongnan Hospital of Wuhan University and approved by the ethics committee (Approval Number 2017058). Written informed consent was obtained from all the participants. The studies were conducted in accordance with the local legislation and institutional requirements. The participants provided their written informed consent to participate in this study.

## Author contributions

SX: Data curation, Investigation, Methodology, Software, Visualization, Writing – original draft, Writing – review & editing. YZ: Methodology, Validation, Writing – review & editing. YY: Methodology, Validation, Writing – review & editing. KD: Methodology, Validation, Software, Writing – review & editing. HZ: Methodology, Software, Validation, Writing – review & editing. CL: Methodology, Writing – review & editing. S-ML: Conceptualization, Funding acquisition, Writing – review & editing.
