# Supplementary material for: A m6A regulators-related classifier for prognosis and tumor microenvironment characterization in hepatocellular carcinoma
Source: Front Immunol. 2024 Jul 25;15:1374465. doi: 10.3389/fimmu.2024.1374465 (PMC11306056; doi:10.3389/fimmu.2024.1374465)

**A m^6^A regulators-related classifier for prognosis and tumor microenvironment characterization in hepatocellular carcinoma**

Shaohua Xu ^1, 2, #^, Ying Yang ^1, #^, Kexin Dong ^1^, Hanfei Zhang ^1^, Chunhua Luo ^2^, Song-Mei Liu ^1, *^

This article contains Supplementary Figures S1-S7 and Supplementary Tables S1-S20.

**Supplementary Figure captions:**

**Figure S1**. The survival analysis of the m6A genes based on TCGA-LIHC & ICGC-LIRI-JP database.

**Figure S2**. Unsupervised clustering of 23 m^6^A regulators in TCGA-LIHC cohort.

**Figure S3.** Determination of soft-threshold power in the WGCNA. (A-B) Scale independence and mean connectivity analysis for various soft threshold powers. The soft-thresholding power was set as 7 for further analysis. (C) Co-expression similarity of all modules based on hierarchical clustering of module eigengenes. The cut height of 0.25 is chosen to merge the similar modules. (D) Module gene-related heat map depicting the topological overlap matrix (TOM) between all modules. Dendograms (upper panel) represent average linkage hierarchical clustering of genes. Increasing color intensity from light color to darker red color corresponds to increasing co-expression-based topological overlap. (E) A scatterplot of (module membership) MM and (gene significance) GS from the blue mod

**Figure S4.** Expression levels of six m6A-related genes of prognostic signature in HCC tissues and corresponding normal tissues by RT-qPCR.

**Figure S5.** Correlations between m^6^Arisk score, tumor associated immune cells and immune phenotypes. (A) Differences in the effector genes of the above tumor-associated immune cells between high- and low-m^6^Arisk score groups. (B) Correlations between m^6^Arisk score and the effector genes of five tumor associated immune cells. The color indicated the Spearman correlation coefficient. The asterisks indicated p-value calculated using Mann-Whitney U test (**P* < 0.05; ***P* < 0.01; ****P* < 0.001). (C) Heatmap showing the representative genes of 122 immunomodulators (chemokines, receptors, MHC, and immunostimulators) with significant differences between the two m6Arisk groups in TCGA-LIHC.

**Figure S6.** Summary of somatic mutation types in the high- and low-m6Arisk score groups. (A-B) The summary of the mutation information, along with statistical calculations in the high- and low-m6Arisk score groups. (C) The quantification results of tumor mutation burden (TMB).

**Figure S7.** Validation and evaluation of nomogram in external cohort. (A) Pooled concordance index for HCC death estimated in the ICGC-LIRI-JP cohort. The C-index was estimated by truncating follow-up time from 1 to 6 years across the entire ICGC-LIRI-JP cohort and plotted on the X-axis as truncated years. (B) Calibration curves of the nomogram in terms of the agreement between predicted and observed outcomes in the ICGC-LIRI-JP cohort. (C-E) The time-dependent ROC curves of the nomograms and clinical characteristics compared for 1-, 2-, and 3-year OS in the entire ICGC-LIRI-JP cohort, respectively. (F–H) The DCA (decision curve analysis) curves showing the comparison of net benefits of the clinical characteristics and the nomogram for 1-, 2-, and 3-year OS in the entire ICGC-LIRI-JP cohort, respectively.

**Figure S1**


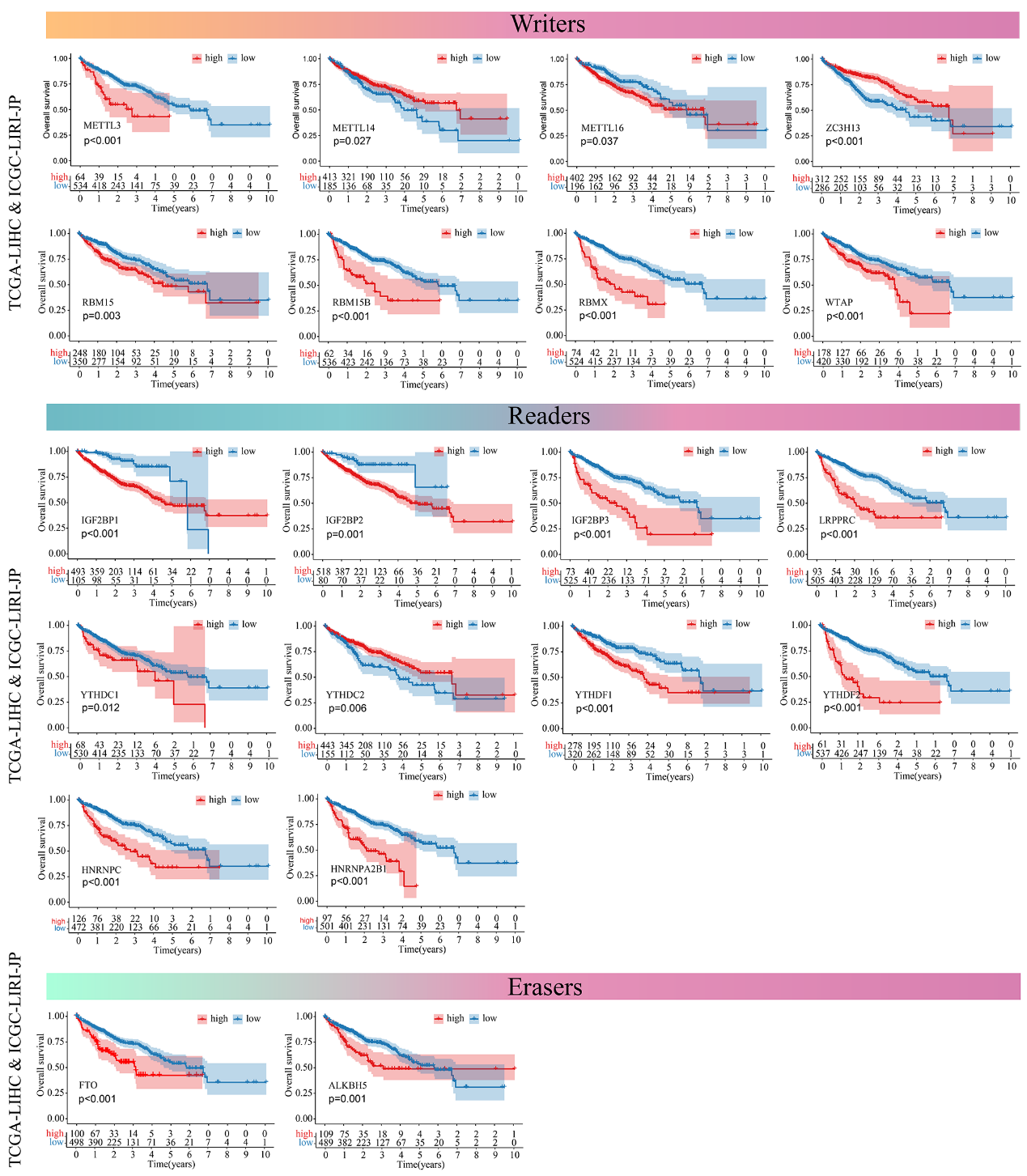


**Figure S2**


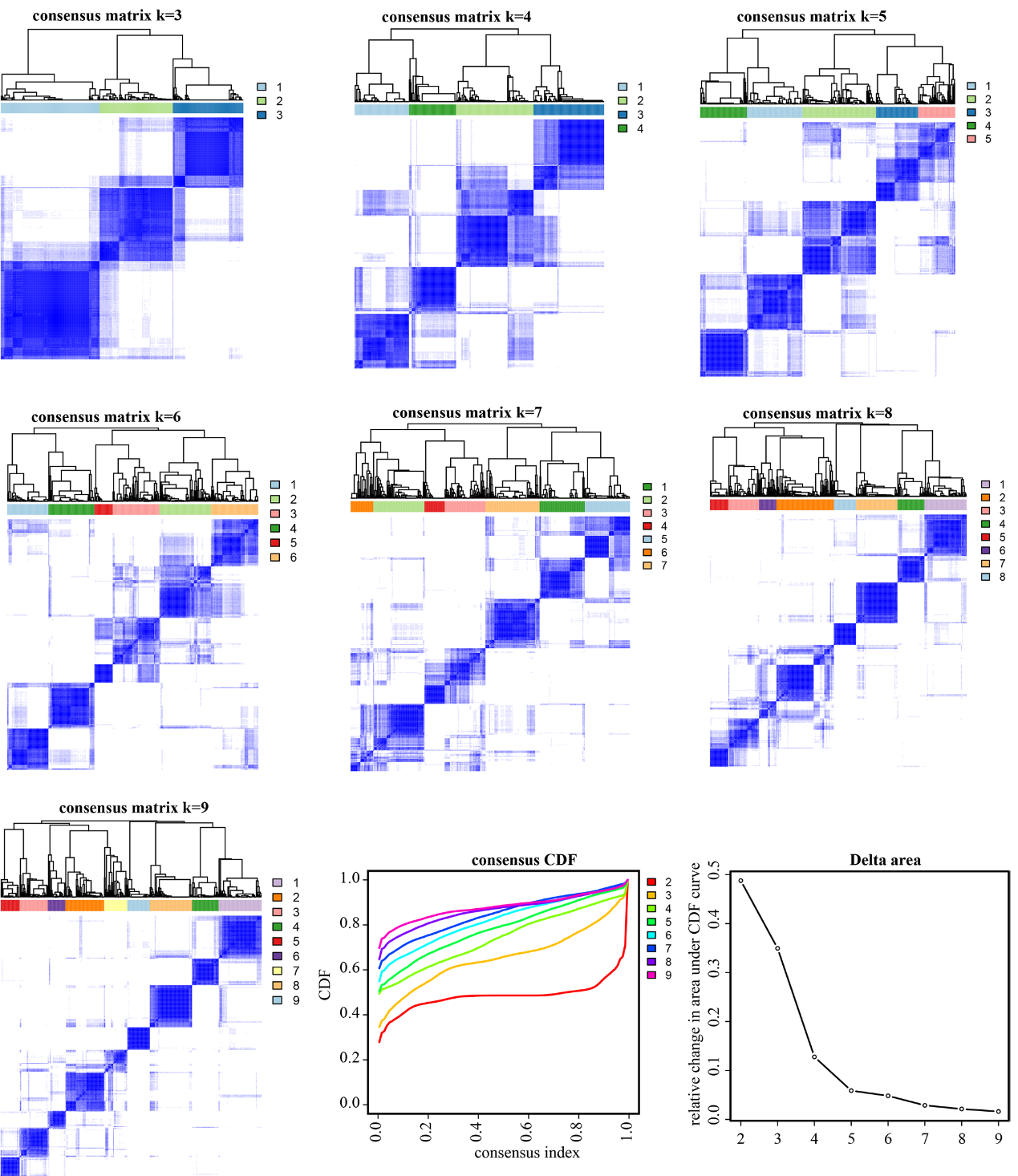


**Figure S3**


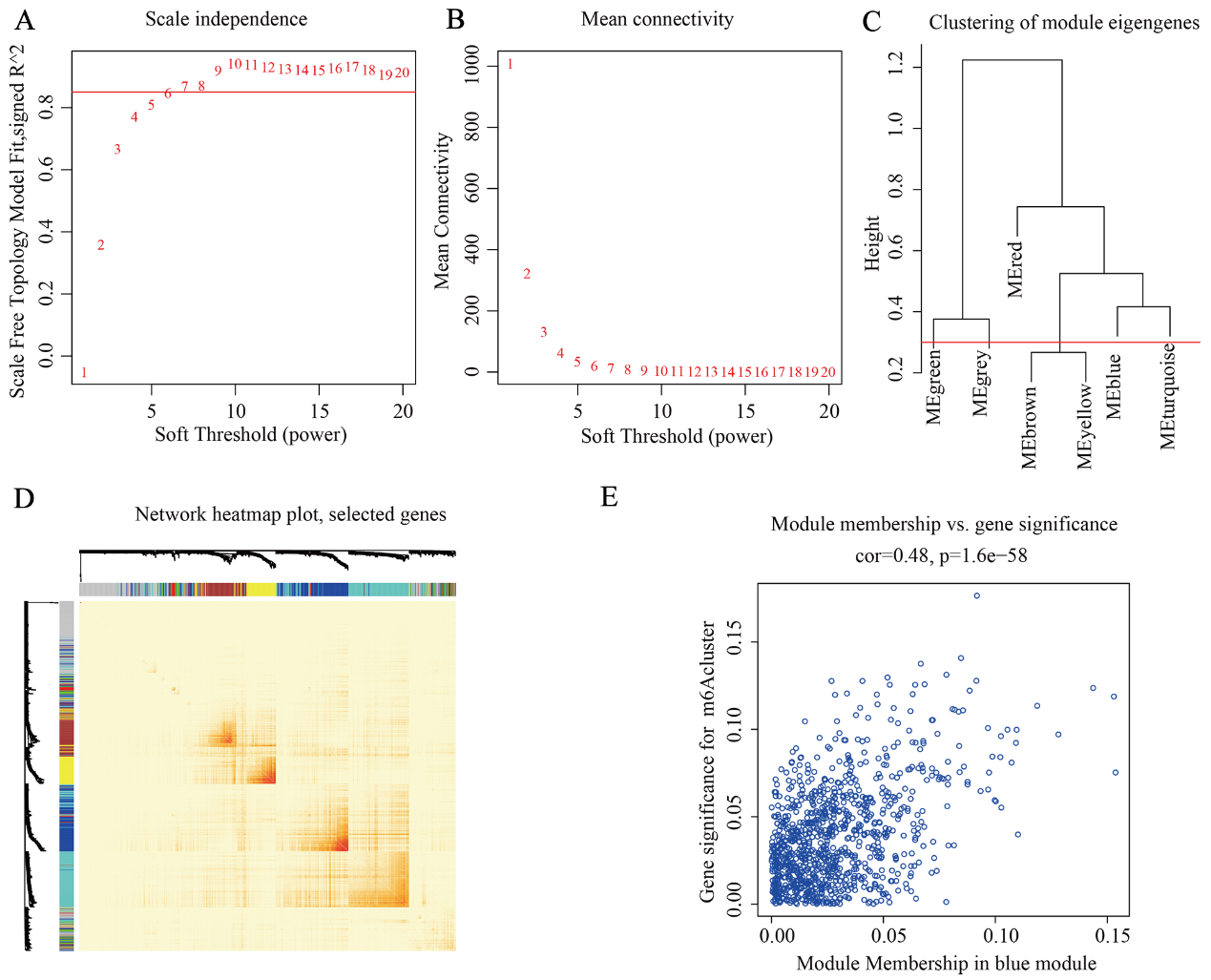


**Figure S4**


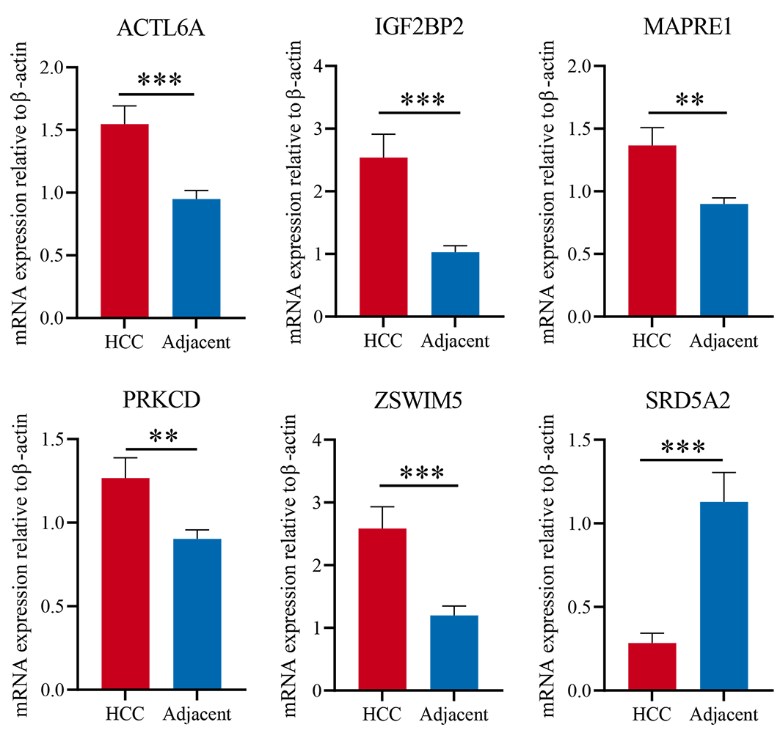


**Figure S5**


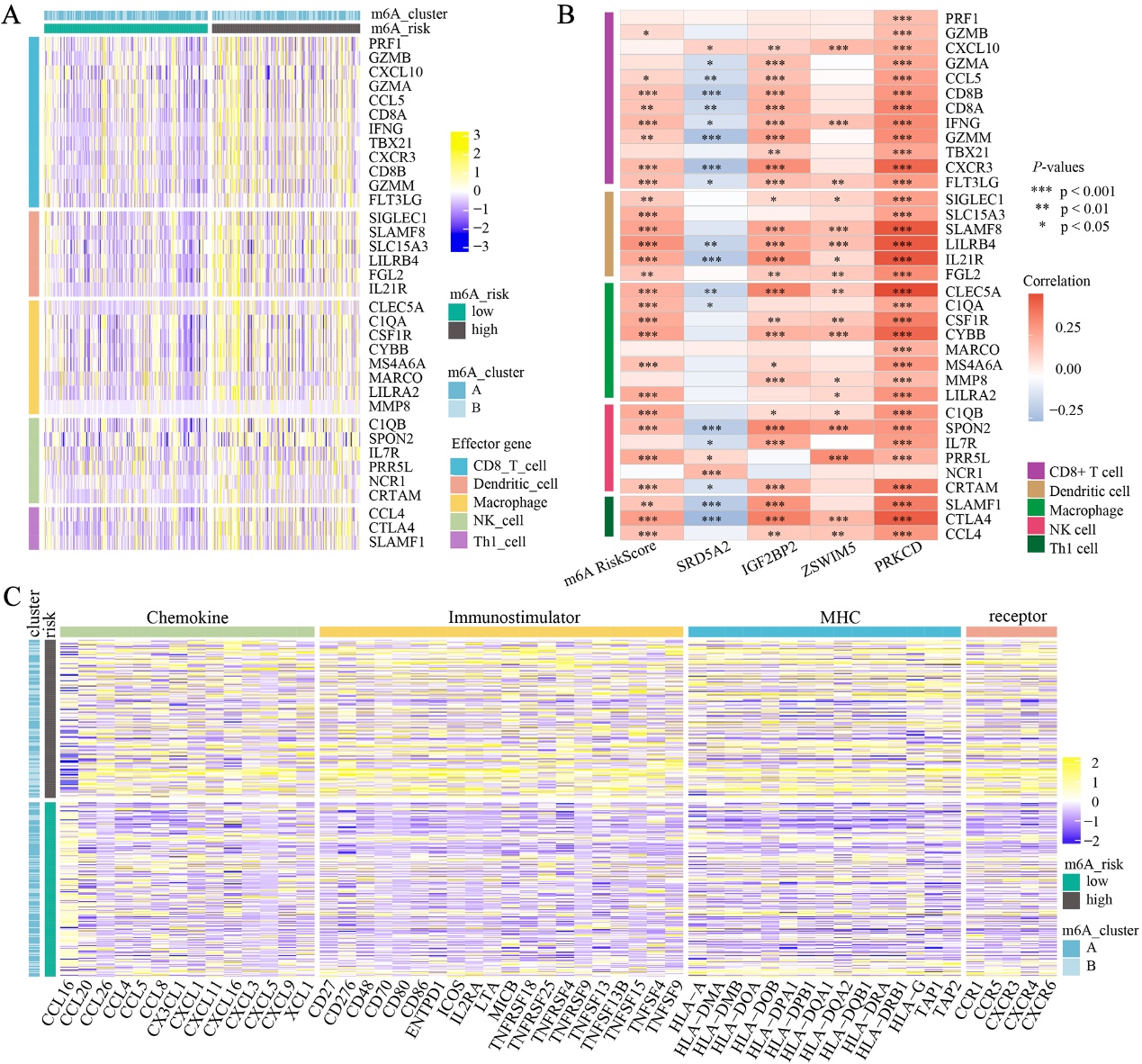


**Figure S6**


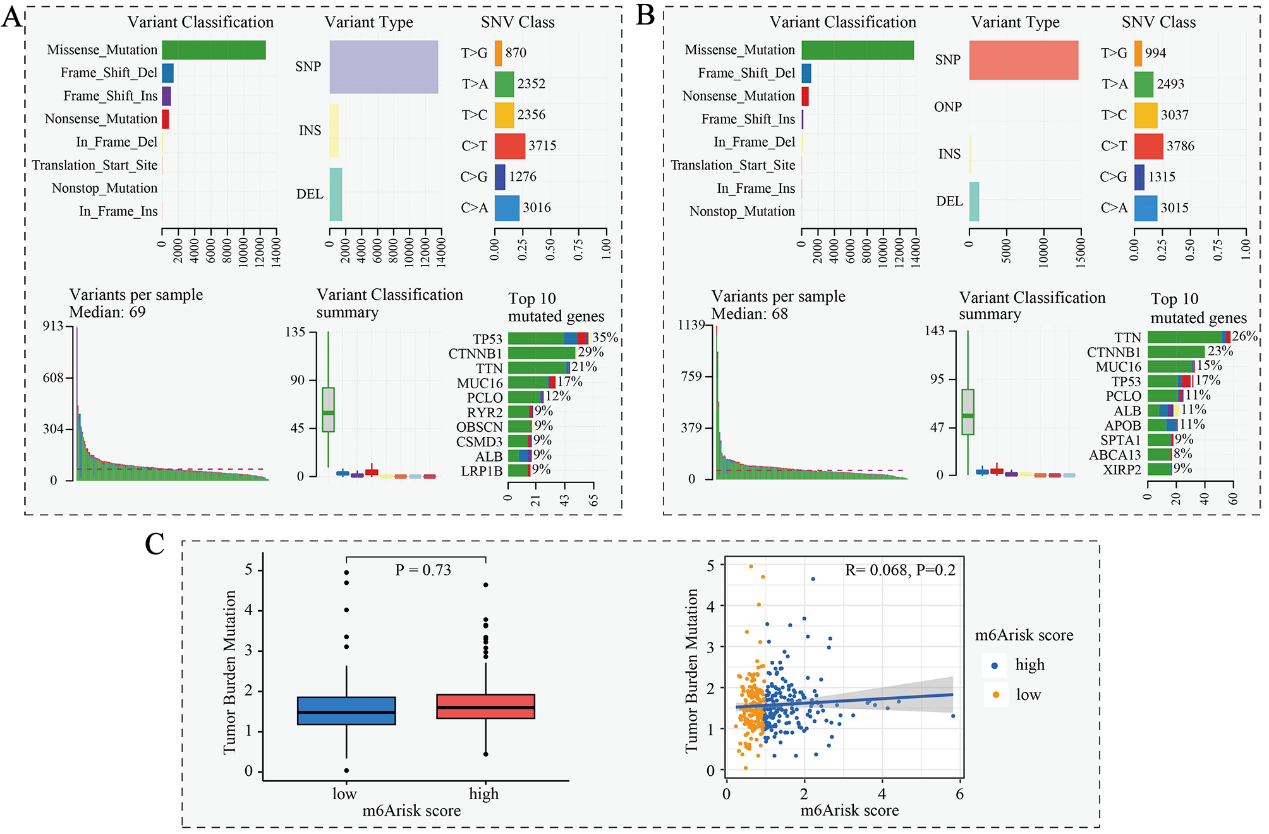


**Figure S7**


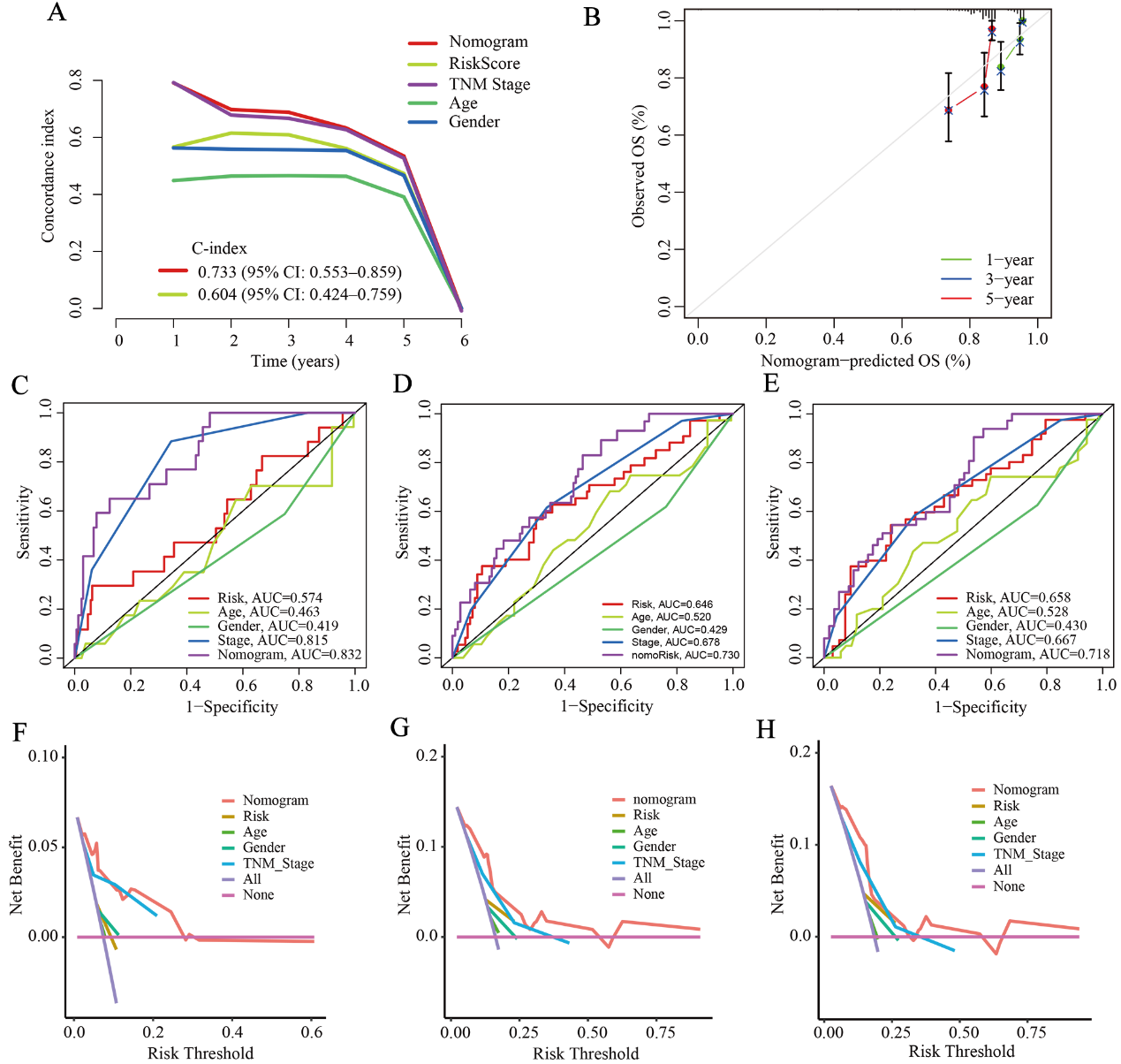

Supplement: Supplementary file 1 [file DataSheet_1.docx]
